# Accelerating Plasmonic Hydrogen Sensors for Inert Gas Environments by Transformer-Based Deep Learning

**DOI:** 10.1021/acssensors.4c02616

**Published:** 2025-01-07

**Authors:** Viktor Martvall, Henrik Klein Moberg, Athanasios Theodoridis, David Tomeček, Pernilla Ekborg-Tanner, Sara Nilsson, Giovanni Volpe, Paul Erhart, Christoph Langhammer

**Affiliations:** † Department of Physics, Chalmers University of Technology, SE-41296 Göteborg, Sweden; ‡ Department of Physics, University of Gothenburg, SE-412 96 Göteborg, Sweden

**Keywords:** hydrogen sensing, plasmonic sensing, nanoparticles, deep learning, neural networks

## Abstract

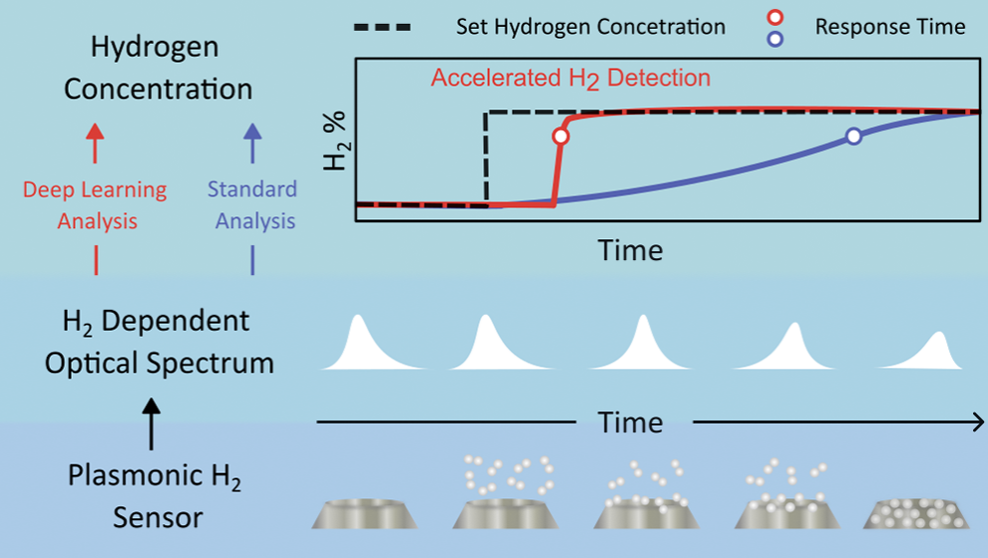

Rapidly detecting hydrogen leaks is critical for the safe large-scale implementation of hydrogen technologies. However, to date, no technically viable sensor solution exists that meets the corresponding response time targets under technically relevant conditions. Here, we demonstrate how a tailored long short-term transformer ensemble model for accelerated sensing (LEMAS) speeds up the response of an optical plasmonic hydrogen sensor by up to a factor of 40 and eliminates its intrinsic pressure dependence in an environment emulating the inert gas encapsulation of large-scale hydrogen installations by accurately predicting its response value to a hydrogen concentration change before it is physically reached by the sensor hardware. Moreover, LEMAS provides a measure for the uncertainty of the predictions that are pivotal for safety-critical sensor applications. Our results advertise the use of deep learning for the acceleration of sensor response, also beyond the realm of plasmonic hydrogen detection.

The ability to detect, quantify, and distinguish chemical species accurately and rapidly is crucial for technologies requiring swift data capture to support well-informed decision-making, automation, and process-monitoring. Such technologies span a wide range of applications, including environmental monitoring,^1^ biosensing for real-time disease diagnostics,^2^ chemical process control^3^ and food quality evaluation.^4^ They all have in common that they critically rely on the development of sensors that are not only precise, sensitive, and selective but also respond rapidly to their target substance and are able to deliver an accurate quantitative measure of the concentration of that target.

A domain that is rapidly expanding and where sensing will play a pivotal role in facilitating safe large-scale implementation is hydrogen-based technologies, including fuel cells for heavy transport, shipping and aviation, energy storage solutions, and green steel production. They all have in common the promise of substantial reductions of greenhouse gas emissions. However, this prospect also generates new demands for active process monitoring and control and introduces safety concerns owing to the high flammability of H_2_-air mixtures. All of these issues can be effectively addressed by the development of accurate H_2_ sensors.

From a sensing environment perspective, two distinct settings exist, where ambient conditions characterized by an abundance of oxygen constitute the most obvious one. The second setting, which is of significant technological relevance but much less discussed in the scientific literature to date, is so-called “inert” or “oxygen-starved” environments. They are established to encapsulate/enclose large-scale H_2_ installations, such as entire engine rooms on fuel-cell-powered ships, or fuel pipes on H_2_-powered airplanes, to avoid the formation of flammable air-H_2_ mixtures. The rapid detection of even the tiniest H_2_ leaks inside these inert gas encapsulation infrastructures is critical to provide enough time for the implementation of appropriate measures to eliminate, as well as spatially localize, the leak by placing sensors at strategic locations inside the system. Specifically, in such installations, the system is continuously flushed by an inert gas, such as N_2_ or Ar, to eliminate or drastically reduce the presence of molecular oxygen. Importantly, we note that the inert gas used in such systems will be of low quality from a purity perspective with respect to species, such as H_2_O, CO, or SO_*x*_, for cost reasons. This combination of lack of O_2_ and the presence of sizable amounts of “poisoning” molecules that bind strongly to many sensor surfaces poses a significant challenge because (i) established H_2_ sensors of the catalytic and thermal type require O_2_ to work and (ii) because the strong molecular bonds either block/poison surface sites required for H_2_ dissociation and/or detection, or facilitate surface reactions that consume hydrogen species and thus prevent them from being detected.^5^

To steer the development of next-generation H_2_ sensors that meet the upcoming demands of the widely implemented H_2_ technologies outlined above, agencies and stakeholders have defined performance targets. The most well-known ones are defined by the U.S. Department of Energy (DOE), which identify sensor speed at ambient conditions as one of the key unresolved metrics.^6^ To this end, a small number of studies exist where H_2_ sensors with response times just below 1 s for a 0.1 vol % H_2_ pulse have been demonstrated experimentally.^7−9^ However, although they are indeed important breakthroughs, these demonstrations were made in an idealized pure H_2_-vacuum environment that constitutes a severe simplification. As the main reason for this simplification, we identify the aforementioned challenge of “poisoning” molecular species in technologically relevant sensing environments due to their impact on the surface chemistry of a sensor. While it has been shown that deactivation-resistant alloys and polymer filters can mitigate sensor deactivation caused by gases such as CO, CO_2_, CH_4_, and NO_2_,^7,10−13^ and that machine learning can help alleviate sensor deactivation due to H_2_O,^14^ the presence of these gases still slows down the sensor’s kinetics. Hence, even though these demonstrations of H_2_ detection with subsecond response in idealized vacuum/pure H_2_ conditions exist, it is clear that further advances in this field are necessary.^5^

Traditionally, such advances are attempted by developing new sensing materials, by nanostructuring the sensing materials and/or signal transducers, and by refinement or modification of fundamental physical sensing mechanisms.^7,9,12,15−18^ Interestingly, however, only limited attention has been directed toward harnessing the potential of tailoring the treatment of output data of existing sensor platforms with the aim to improve the sensor response time, e.g., by machine learning techniques. While several studies have leveraged the potential of machine learning to enhance the *accuracy* or *sensitivity* of different kinds of gas sensors,^14,19−23^ including H_2_, the potential to enhance *sensor response times* has only recently started to gain attention.^24,25^ Specifically, Lin et al. used a convolutional neural network (CNN) to accelerate the response time of a Pd nanocap plasmonic H_2_ sensor, achieving up to a 3.7-fold reduction in response time. While proving the potential of using deep learning for this purpose, there is potential to further accelerate sensing as their CNN-based approach may not capture the full complexity of the sensor’s dynamic behavior.

In response to and motivated by the high demand for faster sensors in general and H_2_ sensors for inert gas environments in particular, here, we develop an approach for accelerating H_2_-sensing that combines optical nanoplasmonic sensors based on hydride-forming metal nanoparticles, such as Pd and its alloys with coinage metals,^5,26,27^ with transformer-based deep learning. As we show below, this combination enables sensor operation in technically highly relevant oxygen-starved environments with significantly improved sensor performance. Specifically, our approach reduces the response time for predicting the H_2_ concentration by up to 40 times in inert gas environments, surpassing conventional methods which are limited by the need to reach full thermodynamic equilibration of the sensor after a change in H_2_ concentration and hampered by sensor deactivation effects due to the presence of molecular contaminants in the inert gas environment. It also provides uncertainty estimates of the sensor response predictions made, which is an important feature for the safety-critical application of H_2_ sensing.

To analyze the output data of plasmonic hydrogen sensors, which typically consists of a time series of scattering or extinction spectra in the visible light spectral range,^5,27^ the current standard analysis (SA) widely applied in the field collapses each such measured spectrum to a single spectral descriptor, such as the spectral peak position, the full-width half-maximum or the centroid position.^28^ As the key point, in this analysis, a significant amount of information contained in both the complete spectrum and its temporal evolution is not used, since it is collapsed into a single descriptor. Hence, what we argue and demonstrate here is that by utilizing *all* of this information via a tailored deep-learning model, it is possible to dramatically improve the sensor performance by analyzing temporal trends of the full spectral information to predict the thermodynamic sensor saturation level *before* this saturation is physically reached and thereby accelerate the sensor response time.

To harness this information with the aim to accelerate plasmonic H_2_ sensor response in general, and in inert gas environments in particular in this work, we introduce LEMAS, short for Long Short-term Transformer Ensemble Model for Accelerated Sensing, which improves the sensor speed by learning the relationship between the time dependence of the full spectrum and the H_2_ concentration, while simultaneously assessing uncertainty in the model predictions through model ensembles. The long short-term transformer (LSTR) architecture consists of long and short-term memory and has been demonstrated to be well-suited for modeling long time sequences.^29^ We demonstrate that LEMAS reduces the response time of a Pd_70_Au_30_ alloy plasmonic H_2_ sensor by up to 40 times when exposed to a distinct H_2_ pulse down to 0.06 vol % H_2_ in an inert gas environment at atmospheric pressure, in a scenario simulating a sudden large leak. Furthermore, we illustrate the ability of LEMAS to rapidly discern and quantify slow gradual changes in H_2_ concentration from mere noise in a simulated scenario of detecting a small leak in an enclosed inert gas environment. This ability is critical for detecting H_2_ at as low concentrations as possible as quickly as possible, allowing sufficient time to apply safety measures, such as system shutdown, before a safety-critical H_2_ concentration is reached. Finally, as an ensemble model, LEMAS enables one to obtain uncertainty estimates, which is of fundamental importance for safety-critical applications, including but not limited to H_2_ sensing. While we focus here on the specific case of hydrogen sensing in an inert environment, we emphasize that LEMAS is broadly applicable to nanoplasmonic sensors in any environment. Our model makes no assumptions about the specific sensing environment or the nanoparticle composition; rather, it is trained on experimental data from a particular sensor particle type obtained in a specific environment as a demonstration. The approach itself, however, is fully adaptable to other sensor types, compositions, and sensing conditions.

## Results and Discussion

### Pd_70_Au_30_ Alloy Plasmonic H_2_ Sensors

**Figure 1:**
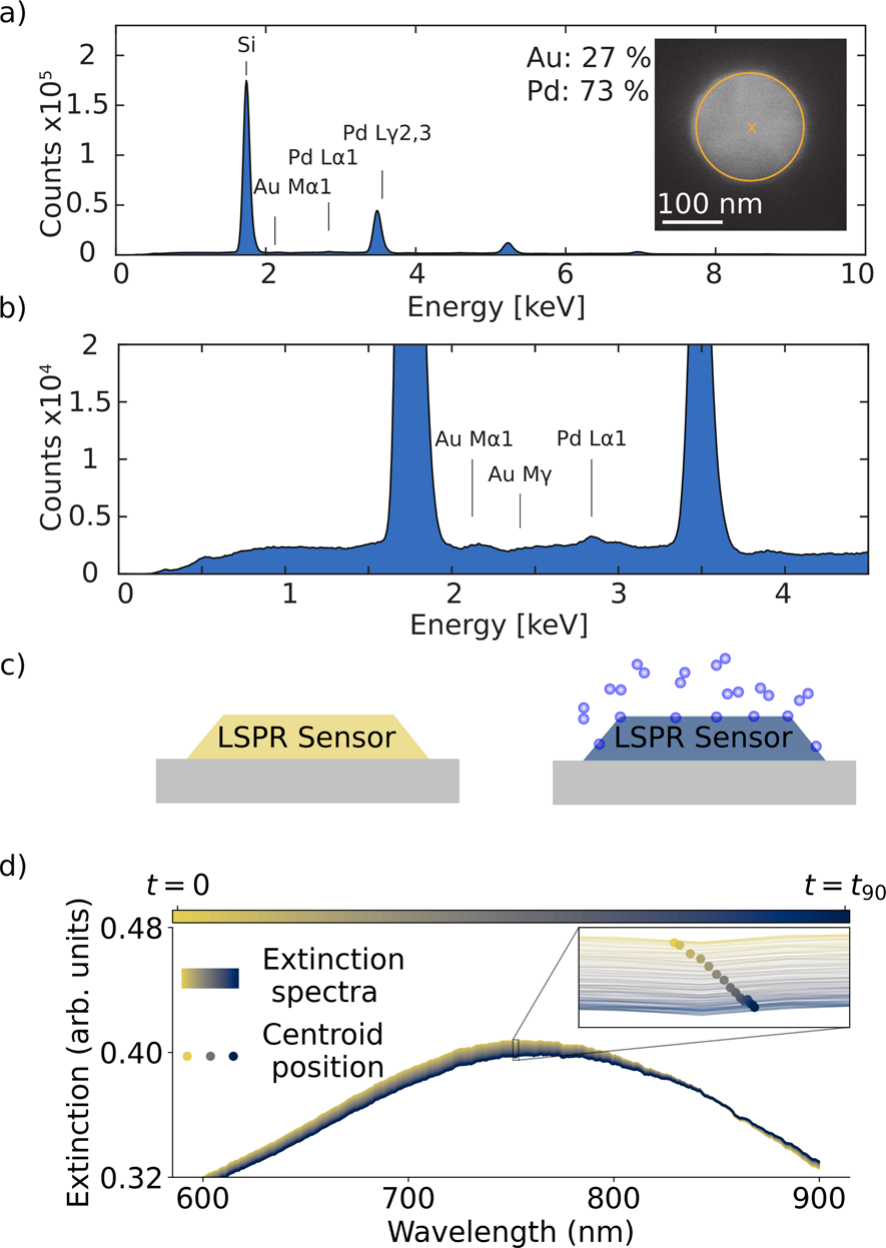
Pd_70_Au_30_ alloy nanoparticle plasmonic sensor characterization and operating principle. (a) Energy-dispersive X-ray (EDX) spectrum collected from a single Pd_70_Au_30_ alloy nanodisk in the quasi-random array of such disks that constitutes the active sensor surface. (b) Zoom-in of the EDX-spectrum in (a) up to 4.5 keV to focus on the characteristic Pd and Au peaks. (c) Schematic illustration of the plasmonic H_2_ sensing principle, where the sorption of hydrogen into hydride-forming metal nanoparticles induces a change in their localized surface plasmon resonance frequency, which leads to a color change that is resolved in a spectroscopic measurement in the visible light spectral range. (d) Example of the spectral response of the Pd_70_Au_30_ alloy plasmonic sensor used in this work, resolved as a gradual shift in the extinction spectrum as hydrogen is absorbed the crystal lattice. Inset: Temporal evolution of the peak centroid position is one of the spectral descriptors that can be tracked to enable real time H_2_ detection.

As the plasmonic H_2_ sensor platform of choice, we selected the well-established Pd_70_Au_30_ alloy system, which we have investigated in detail in earlier works, where parameters such as limit of detection (LOD), response times, and sensitivity have been reported.^7,30−33^ This material system is especially suited for inert gas sensing environments since its sensing mechanism, the interstitial sorption of hydrogen into the lattice of the metal host, does not require O_2_ to be present. The Au alloyant serves the purpose of eliminating the intrinsic hysteresis characteristic for pure Pd by lowering the critical temperature of the system.^34−37^ At 30% Au the best compromise between completely eliminating hysteresis, establishing a linear optical response to H_2_ and maximizing optical contrast per unit sorbed H_2_ is reached. Therefore, we nanofabricated quasi-random arrays of Pd_70_Au_30_ alloy nanodisks with a mean diameter of 210 and 25 nm height onto fused silica substrates using hole-mask colloidal lithography (Figure 1a,b), following the procedures described in detail in our earlier work^38^ and in Sample fabrication in Methods.

The working principle of plasmonic H_2_ sensors is based on the localized surface plasmon resonance (LSPR) phenomenon, which is characteristic of metal nanoparticles irradiated by visible light. In an optical transmission, scattering or extinction spectrum, the LSPR manifests itself as a distinct peak with a maximum at a specific wavelength. The spectral position of this peak maximum, as well as related peak descriptors, such as width and intensity, exhibit a linear dependence on the H_2_ partial pressure surrounding the particles and on the amount of hydrogen species absorbed into interstitial lattice sites of the Pd or Pd alloy host (Figure 1c,d).^32^ Since the absorption and desorption of hydrogen into and from these interstitial lattice positions, respectively, occur spontaneously and reversibly at ambient conditions and also in oxygen-free environments, tracking of the spectral position (as well as other peak descriptors) of the LSPR peak as a function of H_2_ partial pressure enables real-time H_2_ detection (inset in Figure 1d). In this work, for what we refer to as the SA, we use the centroid position as a spectral descriptor, which we relate to the H_2_ concentration by a calibration function (see Standard analysis in Methods and Note S3 for details).

### Deep Learning Model Selection

**Figure 2:**
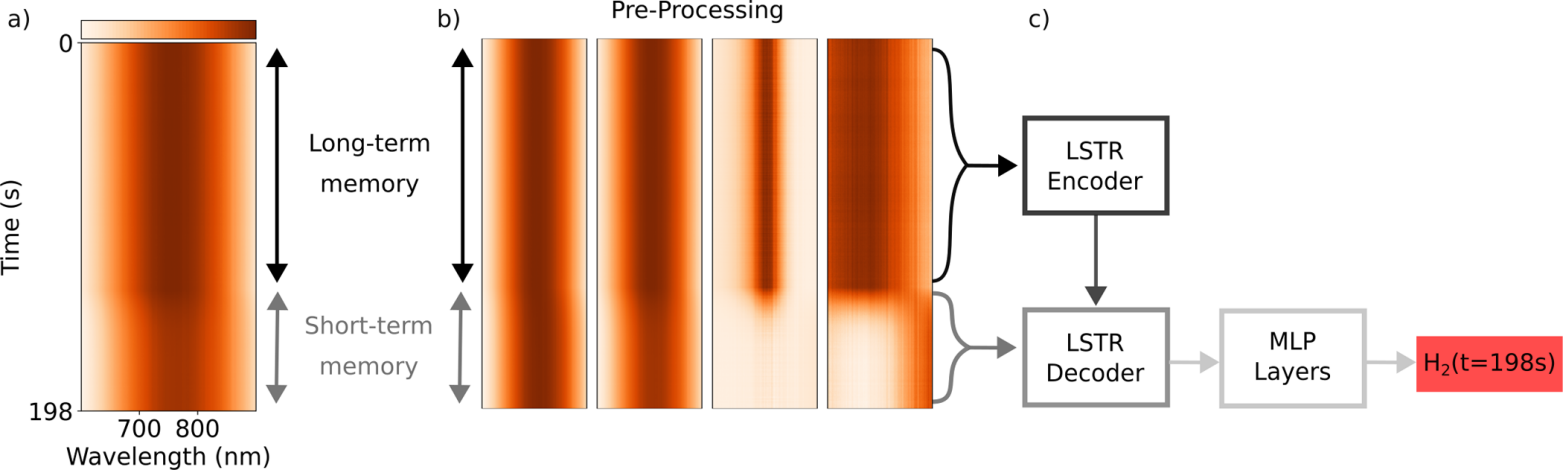
Long Short-term Transformer Ensemble Model for Accelerated Sensing (LEMAS). Illustration of the deep learning model used in this work based on the LSTR architecture (see Figure S16 for a more detailed illustration of the LSTR model). (a) The input data to the model consist of a time sequence of the past evolution of the spectral response of the sensor. In this figure the time sequence consists of 600 time steps corresponding to 198 s. (b) The time sequence is first split into long- and short-term memory and preprocessed using four different methods, including wavelength dependent min-max normalization, standard normal variate standardization, global min-max normalization, and level scaling, before the concatenation of the preprocessed data is being fed to the LSTR. (c) The LSTR first compresses the long-term memory to a fixed length latent representation in the LSTR encoder. Second, the LSTR decoder extracts relevant temporal features in the short term memory while also querying the compressed long-term memory. The extracted temporal features are then passed through a stack of multilayer perceptron (MLP) layers to obtain a prediction of the current H_2_ concentration.

We base our choice of an LSTR model for accelerating the plasmonic H_2_ sensor response on several key characteristics of the output data generated by this type of sensor (see Figure 2 and Deep learning model in Methods for details about the architecture). The first important characteristic to take into account is that the measured extinction spectra that constitute the raw sensor response over time exhibit intrinsic noise (due to intensity fluctuations of the halogen light source and detection noise of the spectrometer used) that is comparable to the magnitude of changes induced in the spectra by small variations in H_2_ concentration. Consequently, a crucial criterion for selecting the deep learning model is its ability to accurately model long temporal sequences, which the LSTR architecture is explicitly designed for.^29^ This capability allows relevant temporal trends in the extinction spectrum to be differentiated from the inherent noise and used for predicting the hydrogen concentration.

The second critical aspect influencing the performance of the LSTR model, based on the characteristics of the sensor data, is the preprocessing of the measured extinction spectra. Such preprocessing is needed due to drift in the sensor response over time (mainly due to long-term variations of light source intensity), as well as small variations in the extinction spectra obtained in different measurements using the same sensor, due to slightly different placement of the sensor in the measurement chamber for each independent experiment. Here, we found that using several preprocessing methods is beneficial for the performance of the LSTR model. Therefore, we used four different preprocessing techniques (see Data preprocessing and Note S2 for details) and concatenated them into a single array.^39^ As a result, the input data for the deep learning models was a time series, where each element in the sequence consisted of the concatenation of the different preprocessing techniques (see Figure 2a).

Another modeling choice that we make is to employ an *ensemble* of LSTR models. This choice is motivated by the safety-critical nature of the hydrogen sensor application and yields a more robust prediction, as well as a measure of uncertainty by aggregating the predictions of several LSTR models to compute the mean and the standard deviation (see Ensembles in Methods for details). Combining these modeling choices, we arrive at LEMAS, characterized by an ensemble of LSTR models that can both rapidly predict the H_2_ concentration and provide a measure of uncertainty from a time series of preprocessed spectra.

### LEMAS Model Training and Testing

Having introduced the architecture of the LEMAS model, we discuss the training and testing data used for optimizing the sensor response in (i) a large and fast leak scenario and (ii) a slow, gradual leak scenario. These data were generated by measuring tailored time series of optical extinction spectra of the sensor localized in a custom-made measurement chamber with a small volume to enable rapid gas exchange at atmospheric pressure to expose the sensor to varying H_2_ concentrations in the simulated inert gas environment as used for encapsulation of hydrogen installations (see Hydrogen sensing experiments in Methods and Figure S1 for details). Specifically, we used three different H_2_ profiles for generating the training data: (i) stepwise increase/decrease of H_2_ from 0.00 vol % H_2_ to 0.06–1.97 vol % H_2_ in inert Ar environment (Figure S4), (ii) linear increase/decrease of H_2_ from 0.06 vol % H_2_ to 0.09–1.97 vol % H_2_ in inert Ar environment (Figure S5a) and (iii) exponential increase/decrease of H_2_ from 0.06 vol % H_2_ to 0.09–1.97 vol % H_2_ in inert Ar environment (Figure S5b), see Note S1 for details.

For the first case of a large simulated leak characterized by a rapid stepwise increase of H_2_ concentration in the sensor surroundings, we trained LEMAS using two measurements of stepwise H_2_ concentration increase/decrease and subsequently tested the trained LEMAS model on a third measurement not used for training. For the second case of a simulated small slow leak, we trained LEMAS on one measurement of linear H_2_ concentration increase/decrease and tested the performance of the trained model on one measurement with exponential H_2_ increase/decreases (see Deep learning training for details).

In this study, all measurements were performed using the same sensor. Consequently, applying either LEMAS or SA to a different sensor of the same type may require retraining, as slight variations in sensor responses can arise due to randomness in the fabrication process. However, in a practical application, for other quality control reasons, it would be imperative that the sensor chips used exhibit identical characteristics across different batches of fabrication. This can easily be achieved by state-of-the-art nanofabrication methods, such as deep-uv photolithography or nanoimprint lithography, which all have their origins in microelectronics, where the aspect of high reproducibility is absolutely critical.

The models trained for optimizing the sensor response in a large and fast leak scenario used a total input sequence length corresponding to the past 3 min, whereas the models trained for optimizing the sensor response in a slow gradual leak scenario used an input sequence length corresponding to the past 22 min of the sensor history. These choices were made based on an analysis of the change in centroid position in the training data and an estimation of the length of the time sequence needed to differentiate the slowest occurring process in the sensor output data from the noise in the measurement (see Figures S10 and S11 for details).

### Accelerating Sensor Response to a Simulated Large Leak in Inert Ar Environment

**Figure 3:**
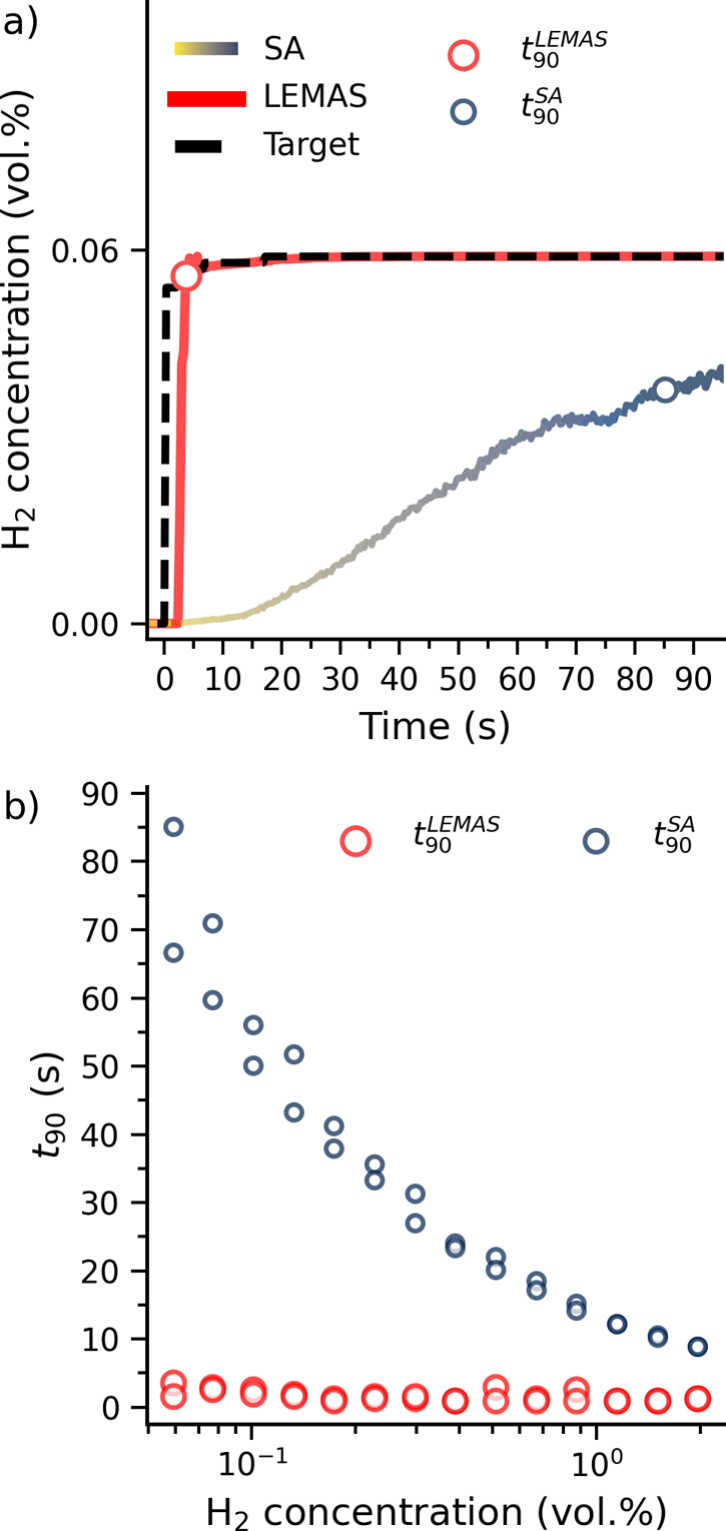
Accelerating sensor response to a simulated large leak in inert gas environment. (a) Comparison of the prediction of LEMAS and the centroid based SA for a pulse of 0.06 vol % H_2_ in inert Ar environment. By utilizing the full time-dependent spectrum of the measured sensor response, LEMAS is able to accurately predict the final value of H_2_ concentration before the sensor physically reaches its new state in equilibrium with the new H_2_ concentration level. (b) Comparison of response times obtained by LEMAS and the centroid based SA as a function of H_2_ concentration in inert Ar environment. Note the significant acceleration by LEMAS, in particular at the lowest H_2_ concentrations, and the elimination of the concentration dependence of the response.

To assess the ability of LEMAS to accelerate the response of a plasmonic H_2_ sensor, we first consider a scenario where a 0.06% H_2_ pulse in inert Ar gas is applied to our device at 30 °C (Figure 3a). For this analysis, we define the response time *t*_90_ as the first point in time where the sensor response has reached 90% of its new steady state value. Applying first the SA that predicts the hydrogen concentration using the instantaneous value of the centroid position reveals that it takes on the order of 85 s to reach *t*_90_ – reflecting the physical time it takes for the response of the system to saturate. Deploying the LEMAS analysis on the same data shows that it is able to predict the saturated H_2_ level, using the *temporal* changes in the extinction spectrum, after only 3.6 s and thus long before the response of the system has saturated, leading to a more than 20-fold reduction of the response time. This result is corroborated when comparing *t*_90_ values obtained by SA and LEMAS across a range of H_2_ pressure pulses from 0.06 vol % H_2_ to 1.97 vol % H_2_ (Figures 3b and S17). While LEMAS demonstrates a significant reduction in response time compared with the SA, it is important to note that the SA approach is computationally more efficient. Fitting the SA model requires only a few seconds, whereas training LEMAS takes approximately 10 h. However, the primary time-consuming aspect for both approaches is not training the model itself but generating the training data; for example, a single measurement requires approximately 20 h (see Figures S4 and S5). Notably, a large number of such measurements would have to be carried out also when using the SA in a practical implementation of the sensor to generate the critically required sensor calibration curve.

Remarkably, LEMAS also achieves a response time that is practically independent of H_2_ concentration whereas the *t*_90_ from the SA quickly increases with decreasing H_2_ concentration. We attribute this behavior to the fact that LEMAS only requires a certain number of data points to identify the change in the extinction spectrum and make its prediction, the availability of which is dictated by the read-out frequency of the spectrometer rather than the H_2_ pressure applied. By contrast, the SA is limited by the intrinsic kinetics of the material platform, causing a strong dependence on the H_2_ pressure. As a result, LEMAS yields the largest boost in acceleration in the application critical range of lower H_2_ pressures, overcoming one of the most important intrinsic limiting factors of hydride-based H_2_ sensors.

Specifically, for the smallest concentrations considered in our experiment, at 0.1 vol % H_2_ and below, the response times range between 1.6–3.6 s for the LEMAS analysis compared to 50–85 s for the SA. This corresponds to a 21- to 40-fold improvement compared to the SA. This improvement exceeds the one achieved by Lin et al., who used a CNN to accelerate a Pd nanocap plasmonic H_2_ sensor, reducing the response time for hydrogen concentrations of 0.1 vol % H_2_ and below to 6–14 s from 13–30 s.^25^ At the same time, we also note that even the accelerated response obtained by LEMAS in the present inert gas conditions is slower than the state-of-the-art in vacuum/H_2_ environment without acceleration.^7−9^ As the main reasons, we identify the following points: (i) The traces of poisoning species, such as H_2_O, CO, etc. present in the Ar inert gas used, significantly decelerate the sensor, as expected^7^ (see Figures S2 and S3 for quantitative mass spectrometric analysis of the background molecular species present in the Ar inert gas used). (ii) We have used relatively large nanoparticles, and it is known that reducing size increases sensor speed due to reduced hydrogen diffusion path lengths.^7^ (iii) We have not applied any polymer coatings, which are known to accelerate sensor response, as well as protect them from the poisoning molecular species present in the inert gas.^7,40^ Importantly, we emphasize that the primary outcome here is not the *absolute* response time achieved by LEMAS but rather the level of *acceleration*. This distinction is important since, given the generic applicability of LEMAS, it suggests that similar acceleration can be achieved for sensors where the intrinsic physical response is faster than the sensor we use here.

Furthermore, we highlight that the amount of response time acceleration that LEMAS can produce depends not only on the obvious intrinsic response speed of the active sensor material (in our case, the PdAu alloy nanoparticles) but also on the sampling rate of the sensor hardware, where a higher sampling rate enables a larger degree of acceleration. For our experiments discussed so far, we have used a sampling frequency of 3 Hz, which was the highest rate enabled by the used spectrometer. Consequently, in this specific implementation, LEMAS has only three data points available to identify a change in the H_2_ concentration in less than 1 s. Crucially, the acceleration observed in the present case is thus not limited by LEMAS but by the underlying materials and read-out of the used light sampling device. In other words, it has the potential to be significantly improved by using a faster spectrometric device.

**Figure 4:**
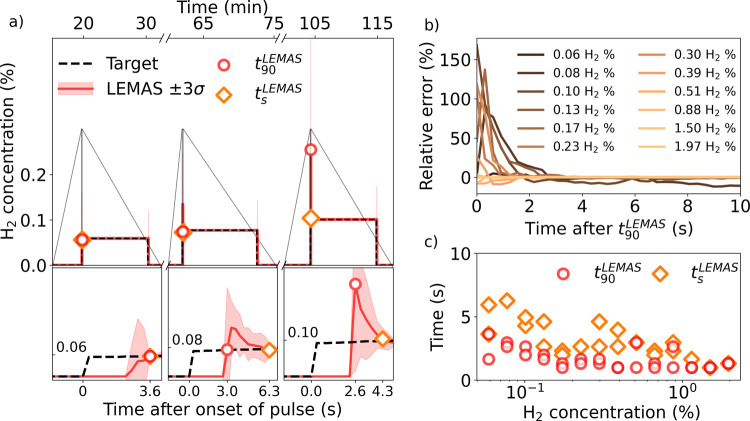
LEMAS prediction accuracy to H_2_ pulses of different concentration. (a) The prediction and standard deviation from LEMAS for three selected H_2_ concentration pulses in the test set. The lower panels display zoom-ins on the initial response to the pulse. (b) The relative error of the LEMAS predictions for the entire range of H_2_ concentration pulses, starting at the response time, , and forward. (c) LEMAS settling and response times as a function of the H_2_ concentration.

Having established the overall LEMAS concept and demonstrated its ability to substantially accelerate sensor speed in inert sensing environments, in particular, in the low concentration regime, it is interesting to evaluate the performance of LEMAS in more detail. To do so, we select three different H_2_ concentration pulses, i.e., pulses to 0.06, 0.08, and 0.10 vol % H_2_, and plot the sensor response predicted by LEMAS as a function of time, with the standard deviation of the prediction at each time point indicated in the corresponding graphs (Figures 4a and S18 for all pulses). We also define the sensor settling time, , as the first time point where the predicted H_2_ concentration lies within ±10% of the target H_2_ concentration and the relative standard deviation is smaller than 10%. This metric complements the response time by also considering cases where LEMAS either underestimates or overestimates the H_2_ concentration after *t*_90_.

First, we note that at the onset of each pulse, there is a brief interval where LEMAS predicts 0 vol % H_2_, while the actual H_2_ concentration has already increased. This behavior occurs since the change in the extinction spectrum induced by the presence of H_2_ is not yet distinguishable from the noise level in the measurement. This initial phase is followed by an interval where a clear change in the extinction spectrum is detected but where both error (Figure 4b) and uncertainty are still rather large (red-shaded areas in Figure 4a). In the final phase, the LEMAS-predicted H_2_ concentration settles at the correct value once the change in the extinction spectrum is sufficiently distinct, such that all models in the ensemble predict a similar H_2_ concentration, and the uncertainty becomes very small.

Finally, we note that for some pulses (illustrated by 0.08 and 0.10 vol % H_2_ in Figure 4a) the predicted H_2_ concentration is overestimated for a brief interval past , before the mean prediction settles around the target value. Consequently,  is larger than . Conversely, in other cases (illustrated by the pulse to 0.06 vol % H_2_) the H_2_ concentration is underestimated until , at which point the uncertainty has also been reduced. As a result,  equals  in this (and similar) cases. To further examine the overestimations we analyze the relative error of the predicted response, starting from  (Figure 4b). Overall the relative error tends to be larger for lower H_2_ concentration pulses since there is a transient overestimation in the LEMAS prediction. Consequently,  is generally larger for lower H_2_ concentrations (Figure 4c). This is likely the consequence of the early predictions being more affected by measurement noise since lower H_2_ concentrations are associated with slower absorption kinetics and smaller changes in the extinction spectrum. An important implication of these initial overestimations is that the accuracy of the sensor's initial response can be compromised if one relies on a single model, providing further evidence for the benefit of using an ensemble model, as we do with LEMAS.

### Improving Sensor Response to a Simulated Small and Slow Leak in an Inert Ar Environment

In a practical application in an inert gas environment, H_2_ sensors are not only required for the rapid detection of large leaks with fast and essentially instantaneous increases of H_2_ concentration but also in scenarios where a small leak will lead to a slow increase in H_2_ concentration in an enclosed environment over time. Technically, this translates into the challenge of being able to discern as quickly as possible a tiny sensor signal from noise. To address this scenario in the LEMAS framework, we define the LOD of a sensor as the minimal amount of H_2_ required for the mean H_2_ prediction to change by more than three times the standard deviation of the H_2_ prediction at a baseline where the H_2_ concentration is kept constant. In other words, the smallest H_2_ required to discern (but not quantify) the presence of hydrogen gas with a confidence of 3σ. Furthermore, we define the limit of quantification (LOQ) as the minimal amount of H_2_ for which the mean relative error of the H_2_ prediction is less than 5%. The mean absolute relative error  is calculated over a time window of 2 min, where *N* is the total number of time steps across the window, *P*_*t*_ is the predicted H_2_ value for time step *t* and *T*_*t*_ is the true H_2_ value for time step *t*.

**Figure 5:**
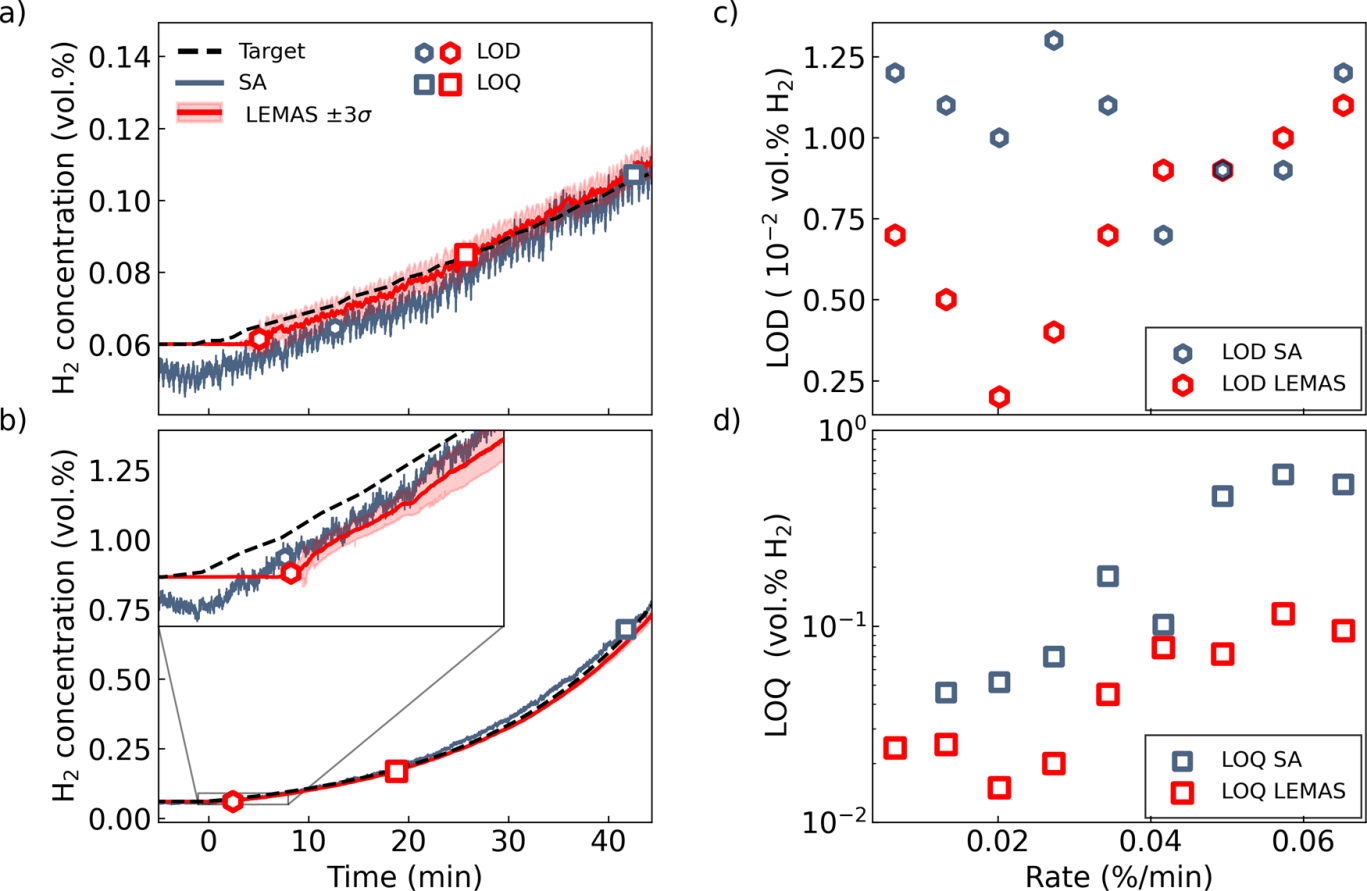
Small leak detection and quantification. (a,b) Time evolution of the H_2_ concentration obtained using the centroid based SA and LEMAS, respectively, for exponentially increasing H_2_ concentration in inert Ar environment for leak rates of (a) 1.32 × 10^–3^ vol % H_2_ min^–1^ and (b) 5.73 × 10^–2^ vol % H_2_ min^–1^. The correspondingly obtained limit of detection (LOD) and limit of quantification (LOQ) are also indicated. (c,d) LOD and LOQ as a function of leak rate. Note that at the smallest leak rate, the LOQ is not reached for the centroid based SA.

We then assess the sensor response to a first scenario with a very small exponential leak rate of 1.32 × 10^–3^ vol % H_2_ min^–1^ using both the centroid-shift-based SA and LEMAS (Figure 5a). This analysis reveals that the LEMAS-predicted H_2_ concentrations contain considerably less noise compared with the SA analysis. In the SA the noise in the H_2_ signal is initially comparable to the change in the H_2_ concentration. As a result, the LOD is reached faster, i.e., at lower H_2_ concentrations, for LEMAS due to its ability to discern changes in the H_2_ concentration. We also note that the LEMAS analysis generally delivers a more accurate response, where the predicted H_2_ concentration values are closer to the target values, resulting in the LOD being reached earlier for LEMAS.

We also perform a similar analysis of a second scenario with a higher exponential leak of rate 5.73 × 10^–2^ vol % H_2_ min^–1^ (Figure 5b; see also Figure S19 for the SA and LEMAS-predicted H_2_ concentrations for all exponential leak rates). Consistent with the previous analysis, LEMAS demonstrates a significantly lower LOQ, which can be attributed to its overall higher accuracy. However, despite LEMAS exhibiting less noise than the SA the LOD of the two approaches is very similar. This can be understood by noting that, in contrast to the previous case, here, the fluctuations in the prediction of the SA analysis are much smaller than the change in H_2_ due to the higher exponential leak rate.

These results are further corroborated when comparing the LOD and LOQ obtained by the SA and LEMAS methods, respectively, across a range of exponential leak rates from 6.52 × 10^–3^ vol % H_2_ min^–1^ to 6.52 × 10^–2^ vol % H_2_ min^–1^ (Figure 5c,d). From Figure 5c we identify that for exponential leak rates at 3.44 × 10^–2^ vol % H_2_ min^–1^ and below LEMAS has a significantly lower LOD than SA. At larger exponential leak rates, the LOD for LEMAS and SA becomes approximately equal. This occurs because, at larger rates, the change in H_2_ is sufficiently large such that the initial change in the sensor signal is much larger than the intrinsic noise. Consequently, the ability of LEMAS to discern small signals from noise does not significantly contribute to decreasing the LOD. At lower leak rates, however, LEMAS indeed makes it possible to extract a discernible signal earlier, at lower leaked concentrations, thereby significantly increasing the time window from triggered sensor response to the leak has reached the flammability limit of 4 vol % H_2_. Finally, in Figure 5d, we see that LEMAS has a lower LOD for all investigated leak rates, which is a consequence of the higher accuracy obtained through LEMAS (see Note S3). In summary, these results underscore, on one hand, the effectiveness of LEMAS in detecting small and slow leaks earlier, as it consistently achieves a lower LOD than the SA at small rates. On the other hand, they demonstrate that LEMAS consistently outperforms the SA in terms of leak quantification, as reflected in its lower LOQ across all rates.

## Discussion and Conclusions

In this work, we leveraged plasmonic H_2_ sensors with deep learning to address the crucial challenge of faster H_2_ detection under technically relevant conditions. We have focused so far on the relatively unexplored yet important application area of inert gas environments intended to enclose large H_2_ installations to avoid the formation of flammable air-H_2_ mixtures. For such applications, hydride-forming plasmonic sensors are ideal, as they do not require molecular oxygen for their operation, unlike the more common catalytic or thermal H_2_ sensors.

From the deep learning perspective, we have developed LEMAS, short for long short-term transformer ensemble model for accelerated sensing, which accelerates sensor response by learning the relationship between the time dependence of the spectral response of the plasmonic sensor and the H_2_ concentration. This allows predicting the sensor’s final response before it is reached physically, while also evaluating uncertainty in the prediction via model ensembles. To obtain accurate models for the ensembles and mitigate artifacts from measurement noise, drift, and variations between different measurements, we found it crucial to use a sufficiently long time series and combine the results of several different preprocessing approaches.

Since LEMAS makes no assumptions about the specific environment or sensor type, the approach developed here can be readily adapted for other sensor types and operating conditions. However, for environments containing higher concentrations of “poisoning” molecular species, appropriate considerations must be made. This requires designing a sensor with deactivation-resistant alloys and/or polymer filters to mitigate the deactivation of the sensor by such molecules. In cases where such solutions are insufficient, we have previously demonstrated how a different transformer-based machine learning approach enables the accurate and stable detection of hydrogen in a highly deactivating environment, i.e., high humidity.^14^ Therefore, we believe that LEMAS could also be applied successfully in such conditions with minor architectural adjustments. The key to reliable application in these scenarios lies in ensuring that the training data for LEMAS are generated under controlled conditions, with systematic variations in the concentrations of “poisonous” molecular species.

Overall, our results demonstrate the significant potential of deep learning to address current H_2_ sensor limitations, such as slow response times and challenges in quantifying H_2_ leaks. This is important from a practical application perspective since it provides a longer time window for the implementation of appropriate measures for handling the leak.

## Methods

### Hydrogen Sensing Experiments

The measurements were conducted in a custom-built reactor chamber that is composed of a customized DN 16 CF spacer flange (Pfeifer Vacuum), equipped with a gas in- and outlet, and two fused-silica viewports (1.33 in. CF Flage, Accu-Glass). The effective chamber volume is ca. 1.5 mL. The gas flow rates were controlled by mass flow controllers (El-Flow Select series, Bronkhorst High-Tech) (Figure S1). The sample inside the chamber was illuminated by using an unpolarized halogen white light source (AvaLight-HAL, Avantes) and an optical fiber equipped with a collimating lens. The transmitted light was collected and analyzed by using a fiber-coupled fixed-grating spectrometer (SensLine AvaSpec-HS1024TEC, Avantes). The temperature was controlled with a heating coil wrapped around the chamber and a temperature controller (Eurotherm 3216) in a feedback loop manner, where the sample surface temperature inside the chamber was continuously used as the input.

All measurements were performed at 30 °C in an argon background, with a constant gas flow of 300 mL/min. The hydrogen concentration in all of the following measurements was in the range of 0.06 vol % H_2_ – 1.97 vol % H_2_ (detailed description of the different pulse schemes as found in Note S1). The sampling frequency of the spectrometer was set to 3 Hz.

### Standard Analysis

In this work, we used the centroid position as a spectral descriptor. The centroid position is defined as λ_c_ = ∑_λ_λ*I*(λ)/∑_λ_*I*(λ), where λ is the wavelength in nm and *I*(λ) is the intensity at wavelength λ. To enable comparison between the SA and LEMAS on the test measurements, we fit a calibration function, using the measured H_2_ concentration in the training measurements, as
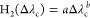
1where Δλ_c_ is the change in centroid position, taken from the smallest centroid position in each measurement. The values of parameters *a* and *b* are determined by minimizing the mean absolute percentage error between the measured H_2_ and H_2_ (λ_c_). We fit two different calibration functions, one for the data consisting of stepwise increase/decrease of H_2_ and one for the data consisting of linear/exponential increase/decrease of H_2_ (see Note S3 for details).

### Data Preprocessing

The spectra recorded in different measurements varied in total intensity due to changes in the light source but also due to slightly different positioning of the sensor chip inside the chamber which causes different particles to be probed at a given measurement (see Figure S14). To address this, we preprocessed the data before it was fed into the deep learning model using four different methods, and the concatenation of these methods was fed as input to the deep learning model. This to ensure that spectra corresponding to the same hydrogen concentration were similar across all measurements. Each measurement was preprocessed individually by using the initial sequence of 5 pulses of 1.97 vol % H_2_, for each measurement, to estimate the minimum/maximum/and mean intensity. The preprocessing methods were (i) wavelength-dependent min-max normalization: for each wavelength subtracting the estimated minimum intensity at the corresponding wavelength and dividing by the difference between the estimated maximum intensity of all wavelengths and the estimated minimum intensity measured at the specific wavelength, (ii) standard normal variate standardization: scaling each spectrum using its mean and standard deviation, (iii) global min-max normalization: subtracting the estimated minimum intensity and dividing by the difference between the estimated maximum and minimum intensity in, and (iv) level scaling: subtracting and dividing each wavelength in each spectrum in the measurement by the estimated mean intensity of each wavelength (see Note S2 for details).

### Deep Learning Model

The deep learning architecture that was used in this work was a long short-term transformer (LSTR)^29^ which operates as illustrated in Figure S16. First, each temporal feature consisting of the concatenation of the preprocessed spectrum is linearly mapped to a vector of size *d*_model_ = 256. Subsequently, positional encoding is added and the data is split into short-term memory and long-term memory. Here, we down-sample the long-term memory using a stride of 4. To process the time series, the long-term memory first undergoes a two-stage memory compression through the LSTR encoder, using a set of learnable token embeddings of dimensions *d*_model_ × *n*_1_ and *d*_model_ × *n*_0_. Here, we used *n*_0_ = 8 and *n*_1_ = 4 and the encoder consisted of 4 transformer decoder units.^41^ Second, the LSTR decoder extracts relevant temporal features in the short-term memory while also querying the encoded long-term memory to retrieve useful information from the history of the sensor. Here, the decoder consisted of 8 transformer decoder units. The extracted temporal features are then passed through *n*_MLP_ = 8 MLP layers of dimension *d*_MLP_ = 512 to obtain H_2_ concentration predictions. Here all the transformer decoder units performed multi-head attention with *h* = 8 heads and *d*_*k*_ = *d*_*q*_ = *d*_*v*_ = *d*_model_/*h* = 32, and the dimension of the MLP inside the transformer decoder units was *d*_ff_ = 512. Furthermore, in the LSTR decoder, masked multihead attention was performed such that during training the H_2_ concentration corresponding to each time step in the short-term memory could be used for supervision during training. We used dropout with a value of 0.1 in all layers, except the last *n*_MLP_ MLP layers. These hyperparameters were chosen based on those in the original paper,^29^ with adjustments including additional MLP layers and an increased number of transformer layers in the decoder, which we found beneficial for reducing the noise in the LSTR’s predictions and a smaller value of *d*_model_ and *h* to reduce computational complexity.

### Ensembles

The constructed ensembles comprised ten models, each varying in the lengths of short-term and long-term memory. This variation was designed to induce diversity in the predictive capabilities of the models in the ensemble. Specifically, two models were designated for each combination of long- and short-term memory lengths, while the total length of the input sequence was the same for all models. For the ensemble tailored for leak detection, the length of the time series was 4000 time steps, while the ensemble model aimed at minimizing the response time used an input sequence of 600-time steps. As we mentioned in LEMAS model training and testing, these choices were based on estimating the time sequence length needed to distinguish the slowest process in the sensor data from measurement noise (see Figures S10 and S11 for details). For both ensembles, the selected lengths for short-term memory were 20, 40, 60, 80, and 100 time steps, respectively.

The values of the short-term memory length were selected based on the idea that the short-term memory should be long enough such that the LSTR decoder can analyze the most recent temporal trends but short enough such that we still utilize the LSTR encoder's ability to summarize important long-term history. The choice of the range for these values is based on our understanding of the kinetics of the underlying physical response of the sensor device studied here. The variation in the length of the memories between the models in the ensembles allows each model to capture slightly different patterns and aspects of the data. The length of the short-term memory controls how much of the recent portion the model can access directly, while the LSTR decoder summarizes the rest of the time series, i.e., the long-term memory. This setup should intuitively allow models with different short-term memory lengths to respond slightly differently to recent changes in the data. By aggregating the predictions across all models, using the mean and standard deviation to compute a prediction and measure of uncertainty, we limit the potential that individual model limitations, such as oversensitivity to noise due to the specific choice of short-term memory, cause inaccurate prediction. Instead, since we average over predictions from models with varied memory configurations, the ensemble is less likely to be biased by the limitations of any single model. Furthermore, the uncertainty estimate obtained from the ensemble offers insight into the prediction’s reliability.

It is important to note that apart from the variation in memory lengths, all models shared identical hyperparameters (see Table S1). To make the prediction of the ensemble more robust to potential outliers, we included only predictions that fall between the first and third percentile to compute the ensemble prediction and uncertainty.

### Deep Learning Training

The models were implemented using TensorFlow^42^ and were trained for 100 epochs on Nvidia A100 graphical processing units using the AdamW^43^ optimizer with weight decay 5 × 10^–5^, a batch size of 128, and mean-absolute-error loss. The learning rate was increased linearly from zero to 5 × 10^–5^ during the first 15 epochs and then decayed to zero following a cosine curve. To analyze the impact of different preprocessing methods we used the first half of the data from measurement Figure S4a,c as training data and the other half as validation data (see Note S2).

For the first case of a large simulated leak characterized by a rapid stepwise increase of H_2_ concentration in the sensor surroundings, we trained LEMAS using the data from measurement Figure S4a,c and used the data from measurement Figure S4b to test the model. For the second case of a simulated small slow leak, we trained LEMAS on the data from measurement Figure S5a and used the data from measurement Figure S5b to test the model. Furthermore, during the training phase, each model in the ensemble was exposed to a distinct subset of the training data, comprising a random 90% of the total data set.

### Sample Fabrication

Quasi-random PdAu alloy (nominal 70:30 at. %) nanodisk arrays with 210 nm average disk diameter and 25 nm height were fabricated using hole-mask colloidal lithography (HCL).^44^ The metals were deposited layer-by-layer via electron beam evaporation onto 1 cm × 1 cm fused silica substrates (Siegert Wafer GmbH). Subsequent annealing was performed at 500 °C for 18 h under a flow of 4 vol % H_2_ in Ar to induce alloy formation. A more detailed description of the nanofabrication procedure can be found in our earlier work.^45^

## Data Availability

Data is available from the authors by reasonable request.

## Supporting Information Available

Experimental details: hydrogenation protocols, quadrupole mass spectrometer measurements, and experimental setup; method details: preprocessing methods, standard analysis information, details on the LSTR architecture and hyperparameters; and results: LEMAS predictions for all stepwise and exponential increases (PDF)
