# Supplementary material for: Accelerating Plasmonic Hydrogen Sensors for Inert Gas Environments by Transformer-Based Deep Learning
Source: ACS Sens. 2025 Jan 7;10(1):376–86. doi: 10.1021/acssensors.4c02616 (PMC11773569; doi:10.1021/acssensors.4c02616)
Supplement: Supplementary file 1 — se4c02616_si_001.pdf [file se4c02616_si_001.pdf]

Supporting Information

Accelerating Plasmonic Hydrogen Sensors for Inert Gas Environments by Transformer-Based Deep Learning

Viktor Martvall[1], Henrik Klein Moberg[1], Athanasios Theodoridis[1], David Tomeček[1], Pernilla Ekborg-Tanner[1], Sara Nilsson[1], Giovanni Volpe[2], Paul Erhart[1,*], and Christoph Langhammer[1,†]

[1] *Department of Physics, Chalmers University of Technology, SE-412 96 Gothenburg, Sweden*
[2] *Department of Physics, University of Gothenburg, SE-412 96 Gothenburg, Sweden*
[*] *erhart@chalmers.se*
[†] *clangham@chalmers.se*

Contents

Supplementary Notes **2**
S1. Hydrogenation protocols . . . 2
S2. Data pre-processing . . . 2
S3. Standard Analysis . . . 2

Supplementary Figures **4**
S1. Experimental setup . . . 4
S2. Gas background quadrupole mass spectrometer measurements . . . 5
S3. Gas background quadrupole mass spectrometer measurements . . . 6
S4. Step wise hydrogenation measurements . . . 7
S5. Linear and exponential hydrogenation measurements . . . 8
S6. Calibration function for step-wise increases/decreases . . . 9
S7. Calibration function applied to the test data . . . 9
S8. Calibration function for linear/exponential increases/decreases . . . 10
S9. Calibration function applied to the test data . . . 10
S10. Time series length for models optimized for accelerating sensor response . . . 10
S11. Time series length for models optimized for leak detection . . . 11
S12. Different pre-processing methods . . . 11
S13. Impact of using different pre-processing methods . . . 12
S14. Difference in spectra from different measurements . . . 13
S15. Difference in spectra from different measurements after pre-processing . . . 14
S16. Schematic illustration of the deep learning architecture used in this work . . . 15
S17. Comparison of LEMAS and SA for step-wise increases in test data . . . 16
S18. Initial prediction of mean and standard deviation by LEMAS for step-wise increases in test data . . . 17
S19. Comparison of LEMAS and SA for exponential leak rates in test data . . . 18

Supplementary Tables **19**
S1. Hyperparameters used for all models . . . 19

Supplementary References **19**

Supplementary Notes

Supplementary Note S1: Hydrogenation protocols

The hydrogenation protocols were as follows: 5 pulses of 1.97 vol.% H_2 concentration were introduced at the beginning of each measurement, in order to activate the nanodisks. For the step wise hydrogen profile (Fig. S4), 3 different measurements were performed which consisted of a logarithmic distribution of H_2 pulses (14 steps increase/decrease, 0.06 vol.% H_2 minimum and 1.97 vol.% H_2 maximum concentration), where the pulse durations were set to 5 min, 10 min and 15 min. The time between pulses was 30 min for all measurements.

For the linear hydrogen profile (Fig. S5a), the H_2 concentration was slowly increased linearly to a maximal concentration. For each of the ramp up/down cycles, the maximum concentrations were: 0.09, 0.13, 0.19, 0.28, 0.41, 0.61, 0.90, 1.33, 1.97 vol.% H_2.The ramp-up/down duration was 1 hour. The maximal concentrations were increasing after each ramping up/down and therefore the hydrogen increase rate was changing as well. For the exponential hydrogen profile (Fig. S5b) the H_2 concentration was increased exponentially to a maximal concentration instead of linearly. The duration of the ramp-up/down was 1 hour and the same maximum concentrations were used.

Supplementary Note S2: Data pre-processing

Due to variations in the spectra from different measurements (see Fig. S12 and Fig. S14), it was necessary to pre-process the data before training a model (see Data pre-processing of the main paper). Initially, we only considered one pre-processing method (wavelength dependent min-max normalization), however, as seen by the blue curve in Fig. S13 this caused errors in the model predictions, which are likely due to the pre-processed spectra for similar H_2 concentration being relatively similar. Sequentially, we therefore added standard normal variate standardization (orange curve in Fig. S13), global min-max normalization (green curve in Fig. S13) and level scaling (red curve in Fig. S13). By using all four pre-processing methods the model accuracy increases substantially.

The reason several pre-processing methods improve the accuracy of the deep learning model, we believe, is that it is difficult to correct for the differences in spectra from different measurements using only one pre-processing method. This because spectra from different measurement differ not only in total amplitude but also slightly in shape (see Fig. S14). This causes spectra from different measurements that correspond to different hydrogen concentrations to overlap at certain wavelength intervals, making it more challenging for the deep learning model learn to distinguish between spectra corresponding to different hydrogen concentrations. However, by introducing various pre-processing methods, which enhance different features of the spectra, the deep learning model is able to learn more robust features which are not as sensitive to these slight variations.

In Fig. S12 and Fig. S15 we illustrate how the different pre-processing methods used alter the spectra. Wavelength dependent min-max normalization scales the data to the interval $[0, 1]$, sharpens the peak of the spectrum and enhances differences between spectra corresponding to different hydrogen concentrations. Standard normal variate standardization centers the spectrum to have zero mean and also accounts for potentially different noise levels by dividing each spectrum by its standard deviation. This pre-processing method enhances the red-shift of the spectrum; however, it also reduces the relative change of the peak position. Global min-max normalization, similar to wavelength dependent min-max normalization scales the data to the interval $[0, 1]$. This method maintains the shape of the spectrum, as it subtracts and divides all wavelengths of the spectrum by the same scalar. Level scaling focuses on the relative difference (per wavelength) of the spectra; however, information about the spectral shape is lost.

Supplementary Note S3: Standard Analysis

To obtain the calibration curve for the step-wise increases we used the measurement illustrated in Fig. S4a. For each pulse, we extracted the steady-state centroid shift as well as the H_2 concentration (Fig. S6). The motivation behind only using the steady-state centroid shift is that a unique mapping between the centroid shift and the H_2 concentration does not exist during the transient state. Then we minimized the mean absolute relative error and obtained $a = 0.334$ and $b = 2.91$ (see Eq. (1)).

In Fig. S6 we illustrate the use of the obtained calibration function for predicting the H_2 concentration on the measurement used for testing LSTR Ensemble Model for Accelerated Sensing (LEMAS) (Fig. S4a). The mean absolute relative error of the standard analysis (SA) for this measurement is 14.73×10^{-3} vol.% H_2, which is higher than the mean absolute relative error of LEMAS being 1.048×10^{-3} vol.% H_2, showing that LEMAS has a higher accuracy.

To obtain the calibration curve for the linear/exponential increases we used the measurement consisting of linear increases/decreases (Fig. S5a). Here, we used the entire measurement to obtain a calibration curve, as the H_2 concentration changes slower than the response time of the system (Fig. S8). Then we minimized the mean absolute relative error and obtained $a = 0.40$ and $b = 2.51$. In Fig. S9 we illustrate the use of the obtained calibration function for predicting the H_2 concentration on the measurement used for testing LEMAS (Fig. S5b). The mean absolute relative error of the SA for this measurement is 13.43×10^{-3} vol.% H_2, which is higher than the mean absolute relative error of LEMAS being 7.4×10^{-3} vol.% H_2, confirming again that LEMAS has a higher accuracy.

Supplementary Figures

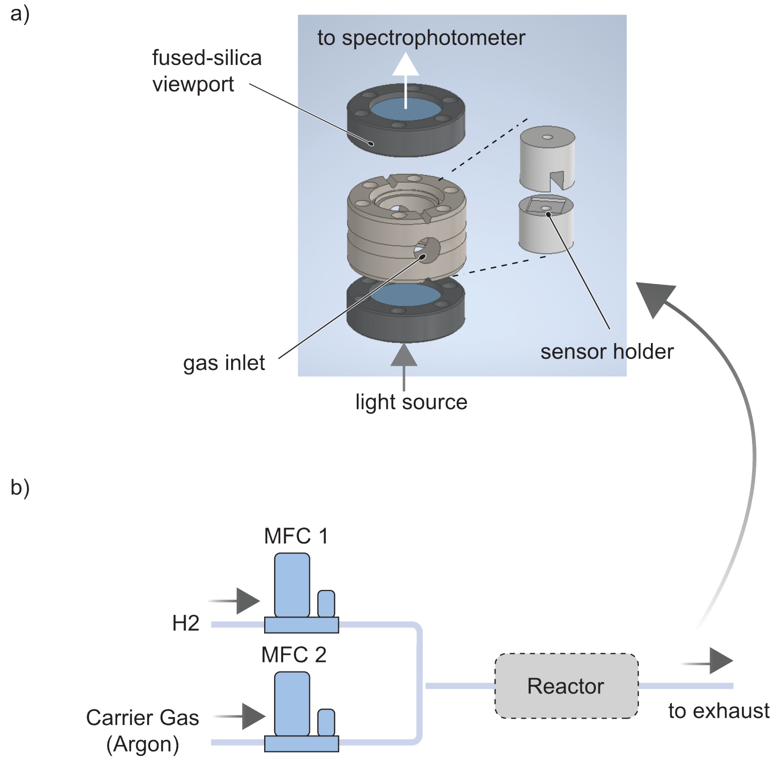

Figure S1: **Experimental setup.** Schematic illustration of the (a) reaction chamber and (b) gas mixing system. Adapted with permission from Ref. 1.

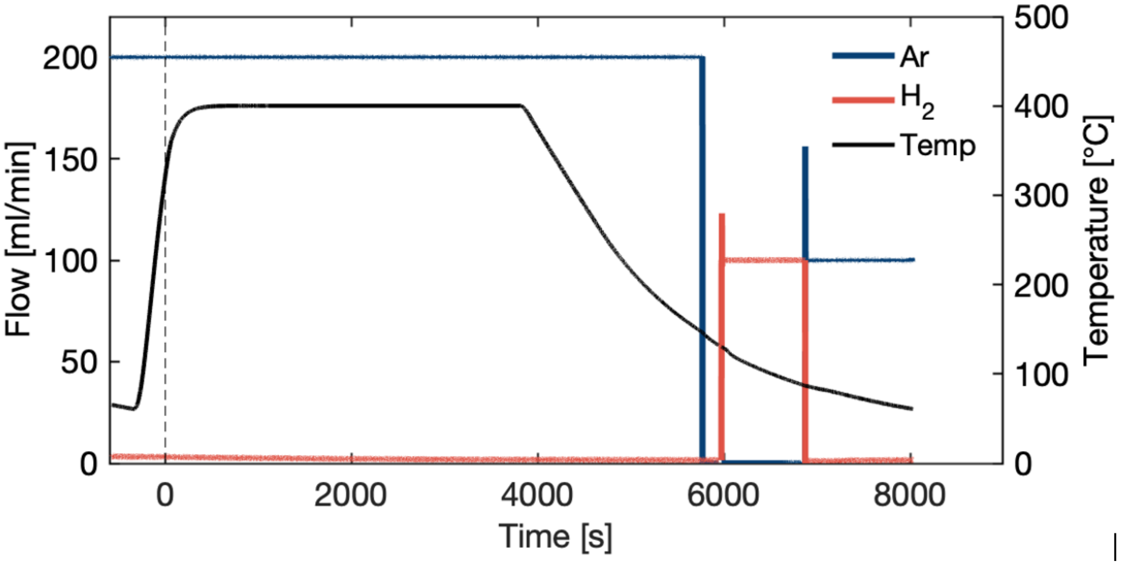

Figure S2: **Gas background quadrupole mass spectrometer measurements.** The gas flows of 100 % Ar (blue, left y-axis) followed by 100 % H_2 (red, left y-axis) and temperature (black, right y-axis) during the quadrupole mass spectrometer (QMS) measurements of the concentrations of possible trace gases in the mixture. The temperature was first increased to 400 °C in 100 % Ar flow, to desorb possible absorbed gases in the flow reactor. This was followed by one 15 minutes 100 % H_2 pulse, and again one pulse of 100 % Ar. The QMS measurement was started at time 0, marked by the dashed line.

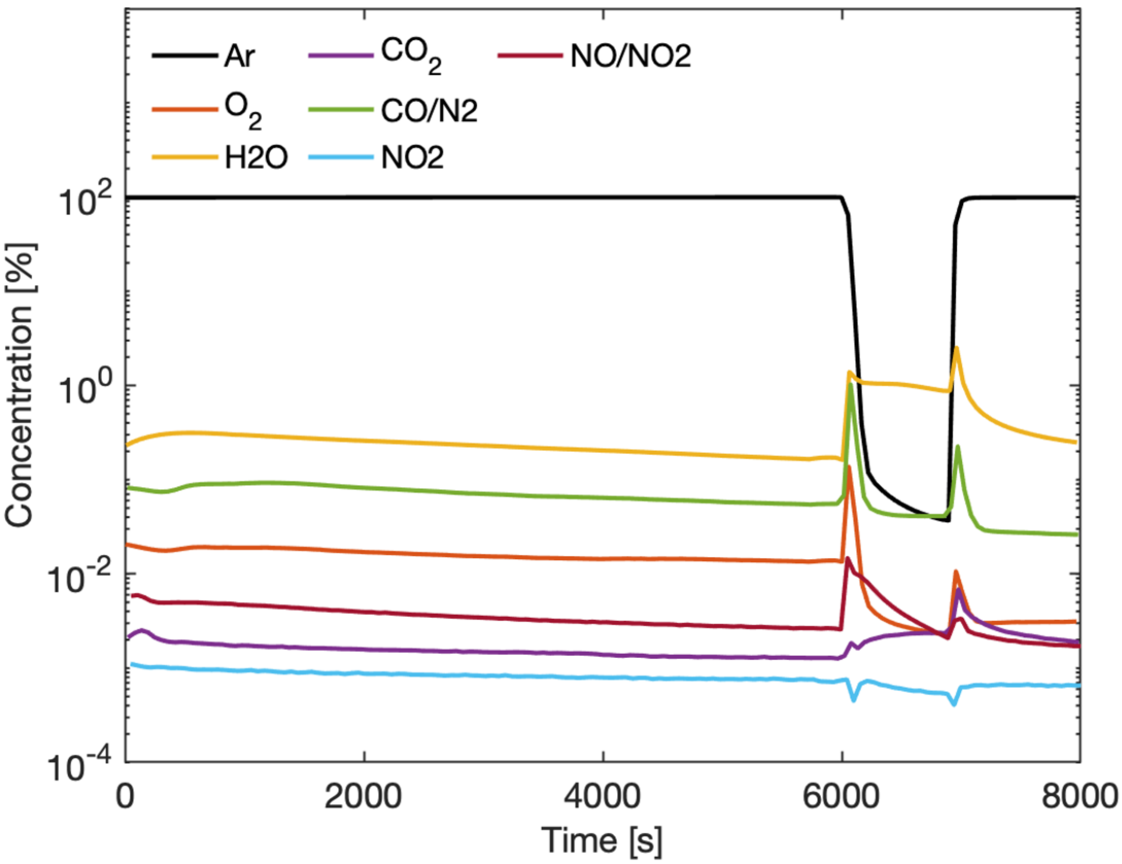

Figure S3: **Gas background quadrupole mass spectrometer measurements.** The concentrations of Ar and the trace gases during the Ar and H2 pulses in Fig. S2. The concentrations of the trace gases at the end of the Ar pulse at 8000 s were 99.7 % Ar, 0.26 % H_2O, 0.027 % CO or N_2, 31 ppm O_2, 18 ppm NO, and 6.6 ppm NO_2.

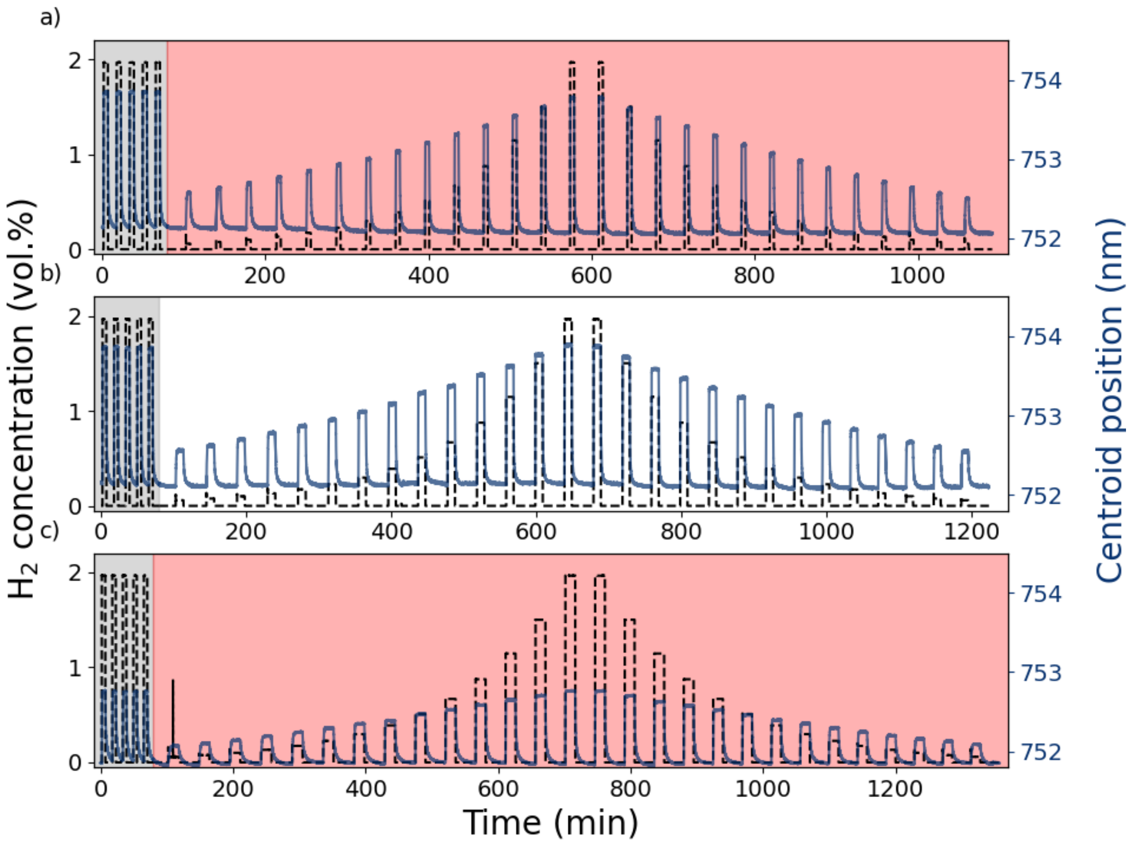

Figure S4: **Step wise hydrogenation measurements.** Hydrogenation protocol for the step wise increase/decrease, alongside the measured centroid shift. (a) 5 min, (b) 10 min, (c) 15 min of pulse duration followed by 30 minutes between each pulse for all measurements. The spectra collected during the first 5 pulses to 1.97 vol.% H_2 (gray shaded area) were used to pre-process the subsequent spectra in each measurement and were not used for training or testing. The measured spectra and set hydrogen concentrations from the red shaded region in measurement (a) and (c) were used for training/validation, with the first 14 pulses being used for training and the last 14 pulses for validation. The measured spectra and set hydrogen concentrations from non-shaded region in measurement (b) were used for testing.

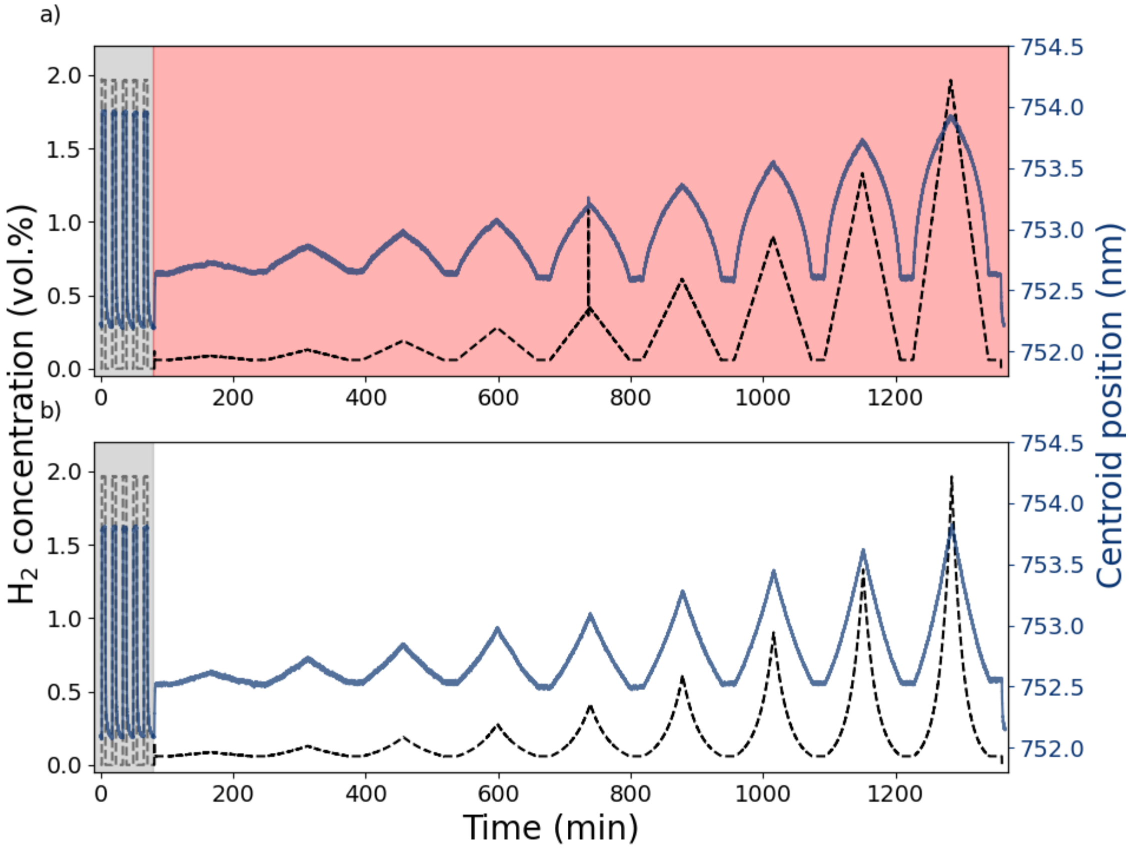

Figure S5: **Linear and exponential hydrogenation measurements.** Hydrogenation protocol for the (a) linear and (b) exponential increase/decrease alongside the measured centroid shift. The ramp-up/down duration for each cycle was 1 h. The spectra collected during the first 5 pulses to 1.97 vol.% H_2 (gray shaded area) were used to pre-process the subsequent spectra in each measurement and were not used for training or testing. The measured spectra and set hydrogen concentrations from the red shaded region in measurement (a) were used for training, while the measured spectra and set hydrogen concentrations from non-shaded region in measurement (b) were used for testing.

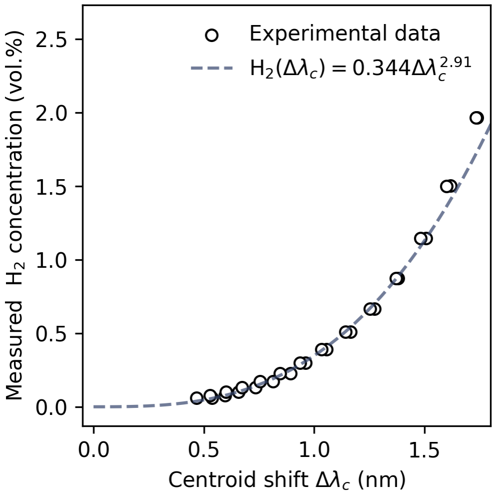

Figure S6: **Calibration function for step-wise increases/decreases.** Experimental data used from measurement Fig. S4a to fit the calibration function for the step-wise increases and the obtained calibration function.

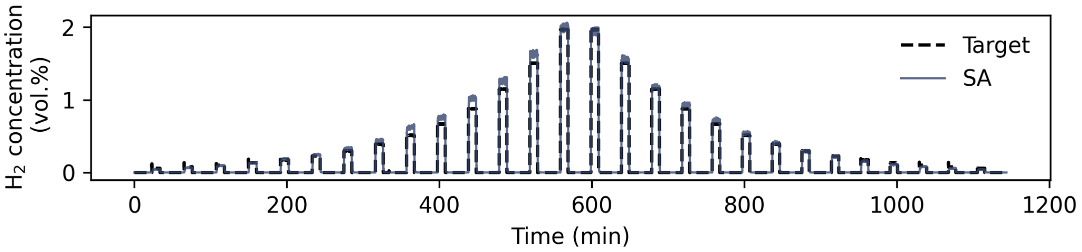

Figure S7: **Calibration function applied to the test data.** Calibration function obtained from Fig. S6 used on the test data, measurement Fig. S4b.

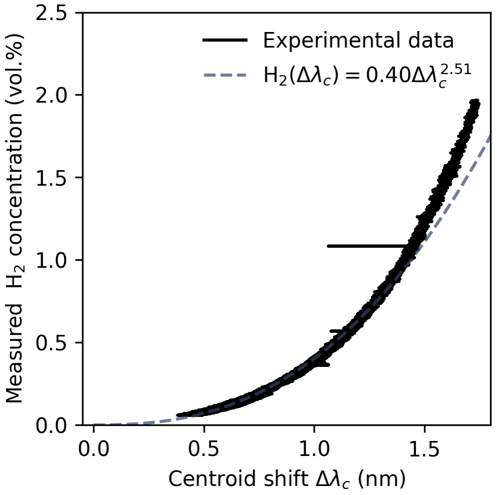

Figure S8: **Calibration function for linear/exponential increases/decreases .** Experimental data used from measurement Fig. S5a to fit the calibration function for the linear/exponential increases and the obtained calibration function.

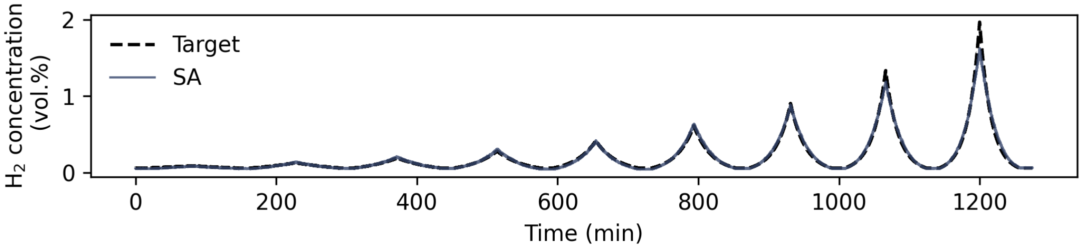

Figure S9: **Calibration function applied to the test data.** Calibration function obtained from Fig. S8 used on the test data, measurement Fig. S5b.

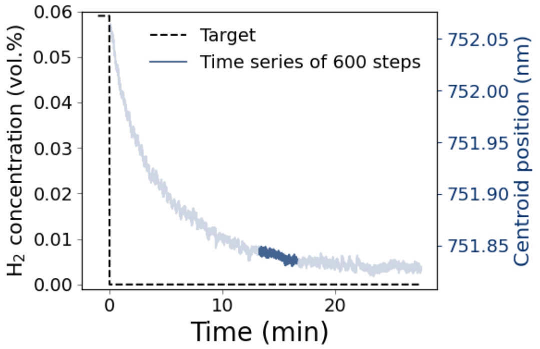

Figure S10: **Time series length for models optimized for accelerating sensor response.** Change in centroid position during the slowest occurring process in the training data, namely the desorption from 0.06 vol.% H_2 in measurement Fig. S4c. A time series comprising 600 time steps is chosen here to allow the model to estimate the rate of change in the spectrum, which is a prerequisite for accurately describing this process.

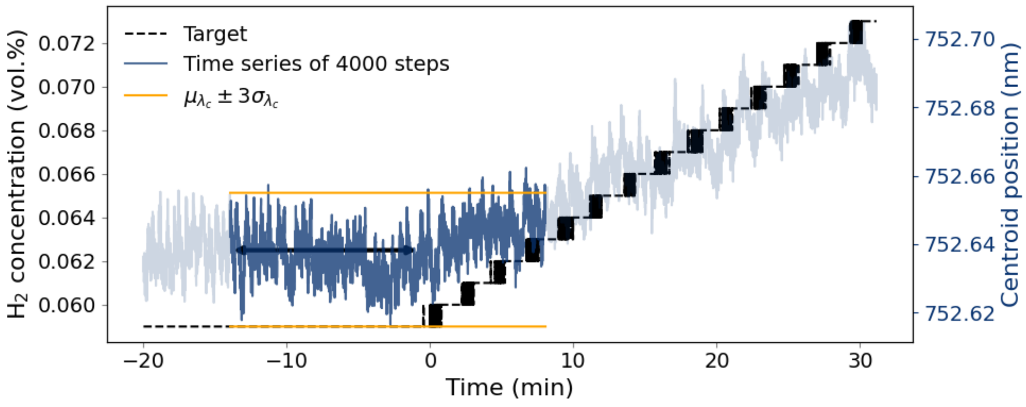

Figure S11: **Time series length for models optimized for leak detection.** The change in centroid position in the beginning of the leak with the smallest leak rate in Fig. S5a, compared to the mean and standard deviation of the centroid position before the leak has started (indicated by the double-headed arrow). Through using a time series comprising 4000 time steps the model should, in the worst case, be able to differentiate the leak occurring with the smallest slope from the noise in the sensor output data approximately 10 min after the leak has started.

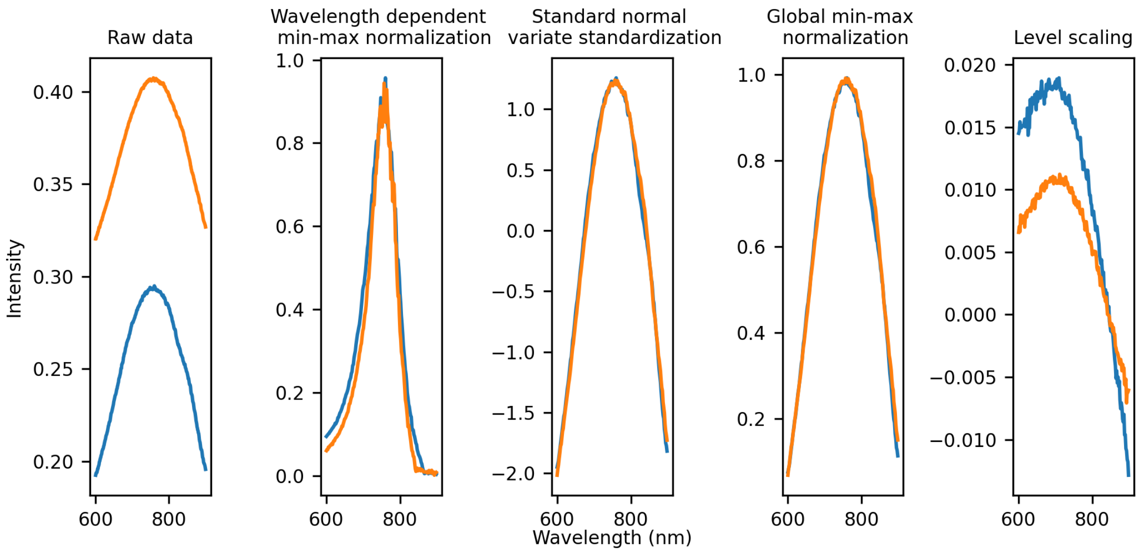

Figure S12: **Different pre-processing methods.** Illustration of the effect of different pre-processing methods on model prediction as well as raw data for spectra from measurement Fig. S4a (blue) and Fig. S4c (orange) at 0 vol.% H_2.

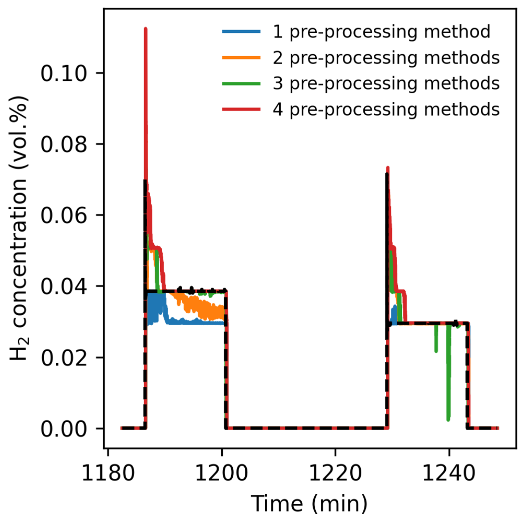

Figure S13: **Impact of using different pre-processing methods.** Predicted H_2 concentration on the two last step-wise increases in measurement Fig. S4c for models trained on the first half of measurement Fig. S4a and Fig. S4c, using different combinations of pre-processing methods.

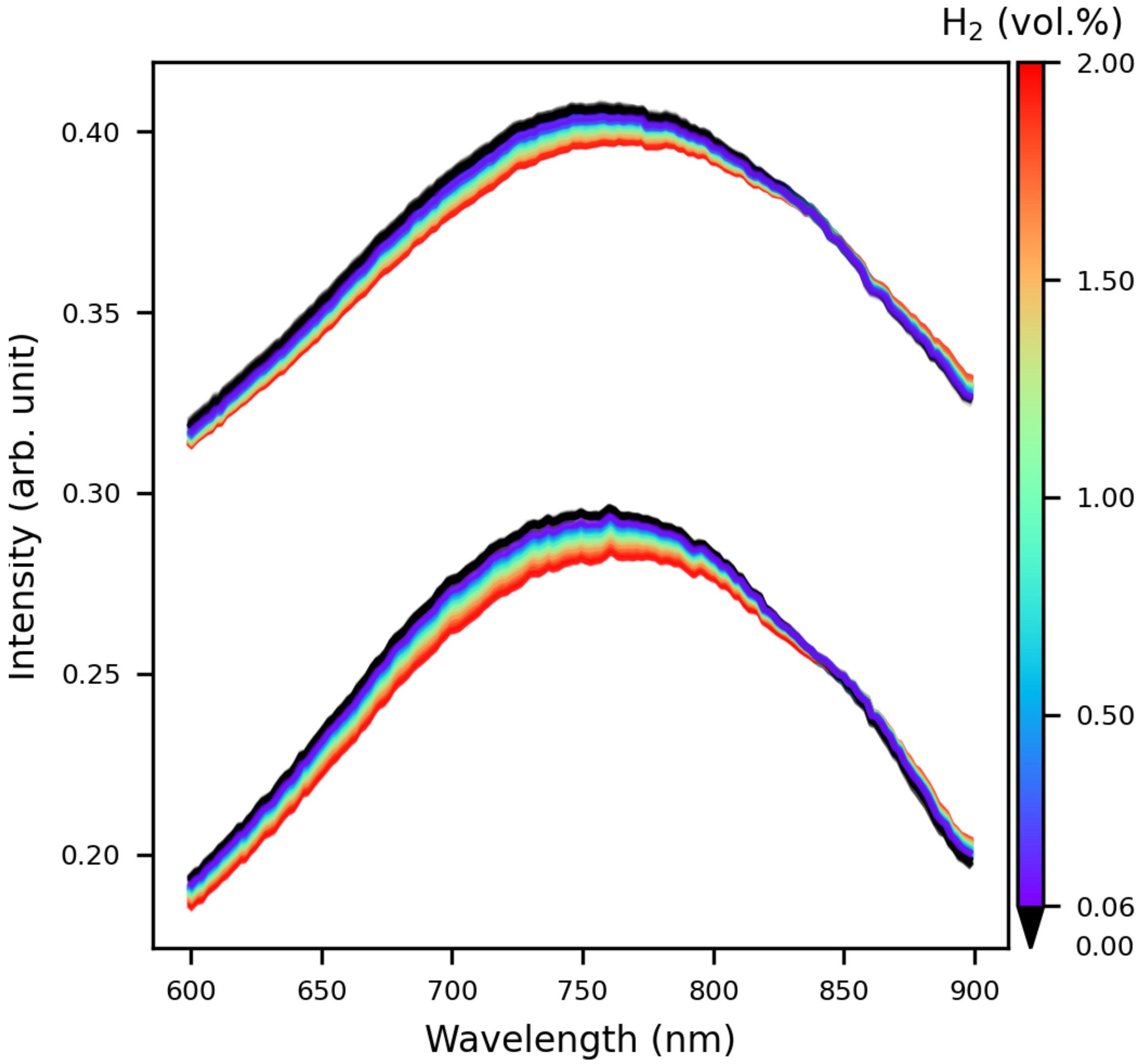

Figure S14: **Difference in spectra from different measurements.** Illustration of spectra from measurement Fig. S4a and Fig. S4c as function of the hydrogen concentration. The spectra differ in total intensity, where spectra from measurement Fig. S4c higher intensity, while the spectra from measurement Fig. S4a has lower intensity. The spectra also differ slightly in shape, most prominent around 850 nm.

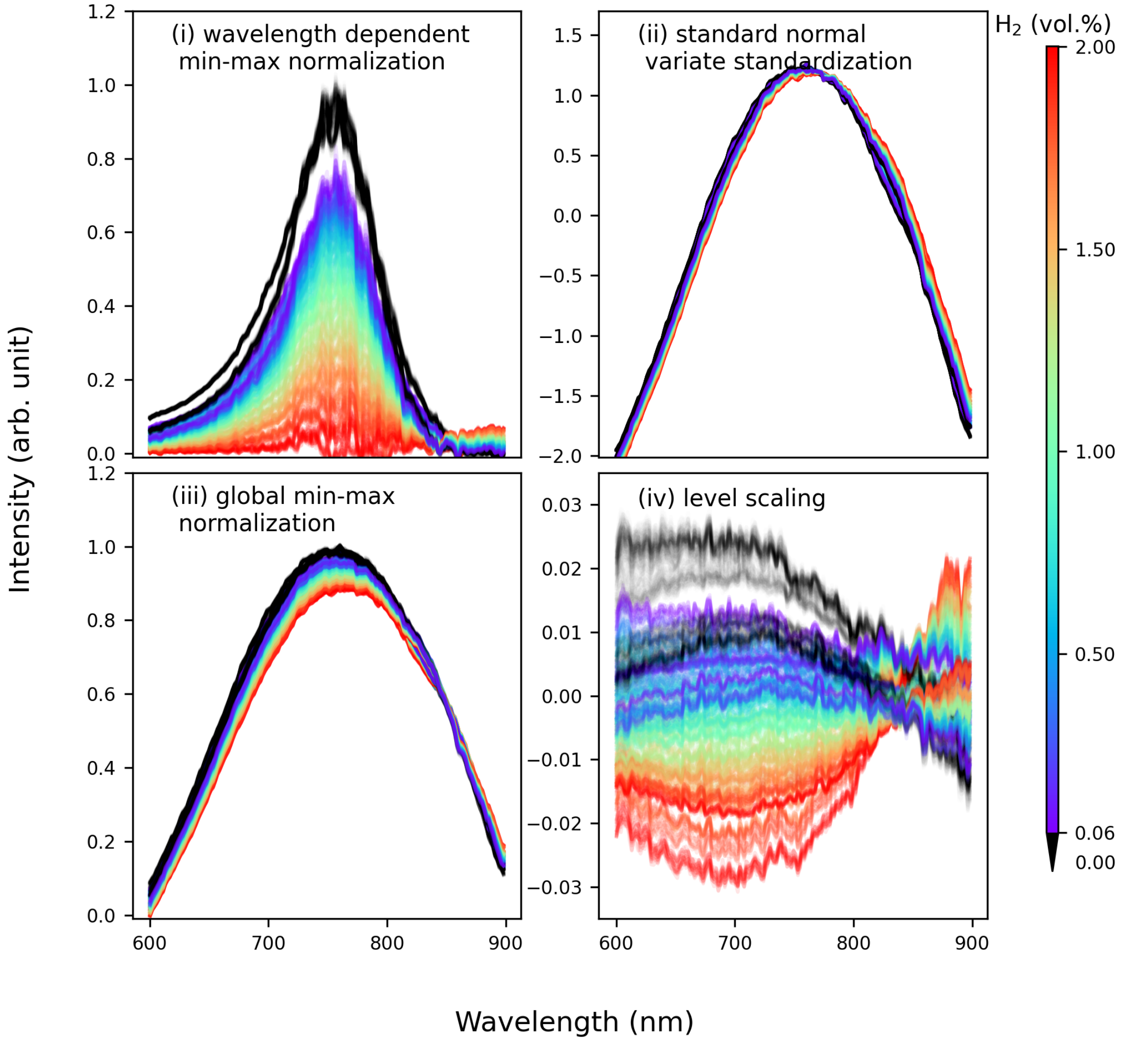

Figure S15: **Difference in spectra from different measurements after pre-processing.** Illustration of spectra from measurement Fig. S4a and Fig. S4c as function of the hydrogen concentration after they have been pre-processed. Comparing with Fig. S14 the difference between the two measurements are smaller, however some disparity still exists, which can be seen by e.g, noticing the two distinct black lines in (i) or (iv) corresponding to spectra at 0 vol.% H_2 in measurement Fig. S4a and Fig. S4c respectively.

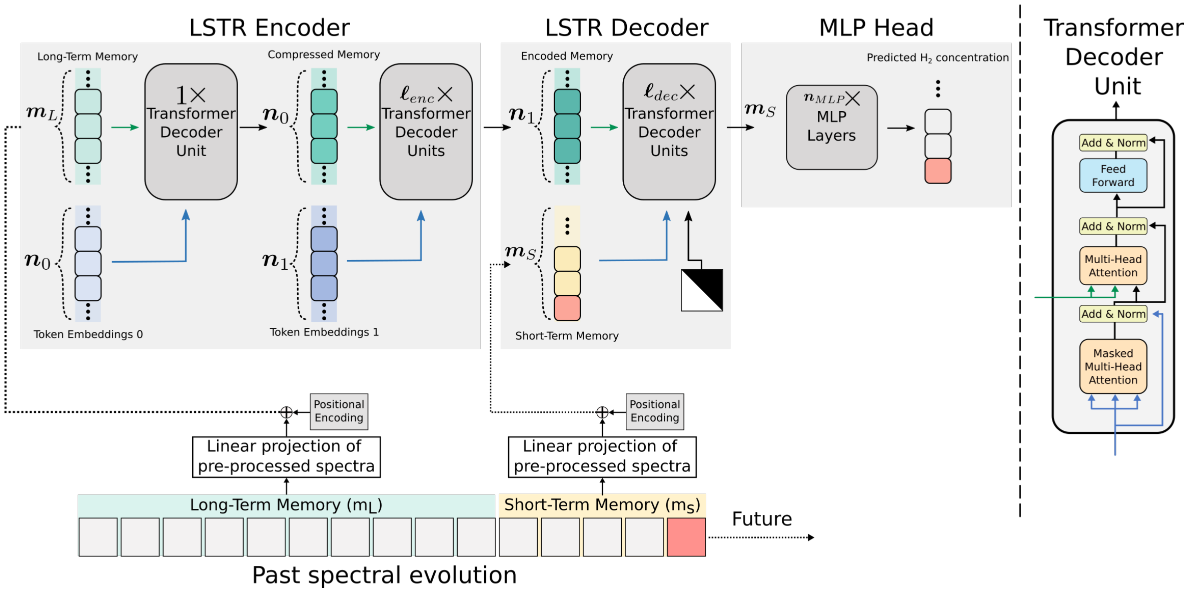

Figure S16: **Schematic illustration of the deep learning architecture used in this work.** Adapted from Ref. 2.

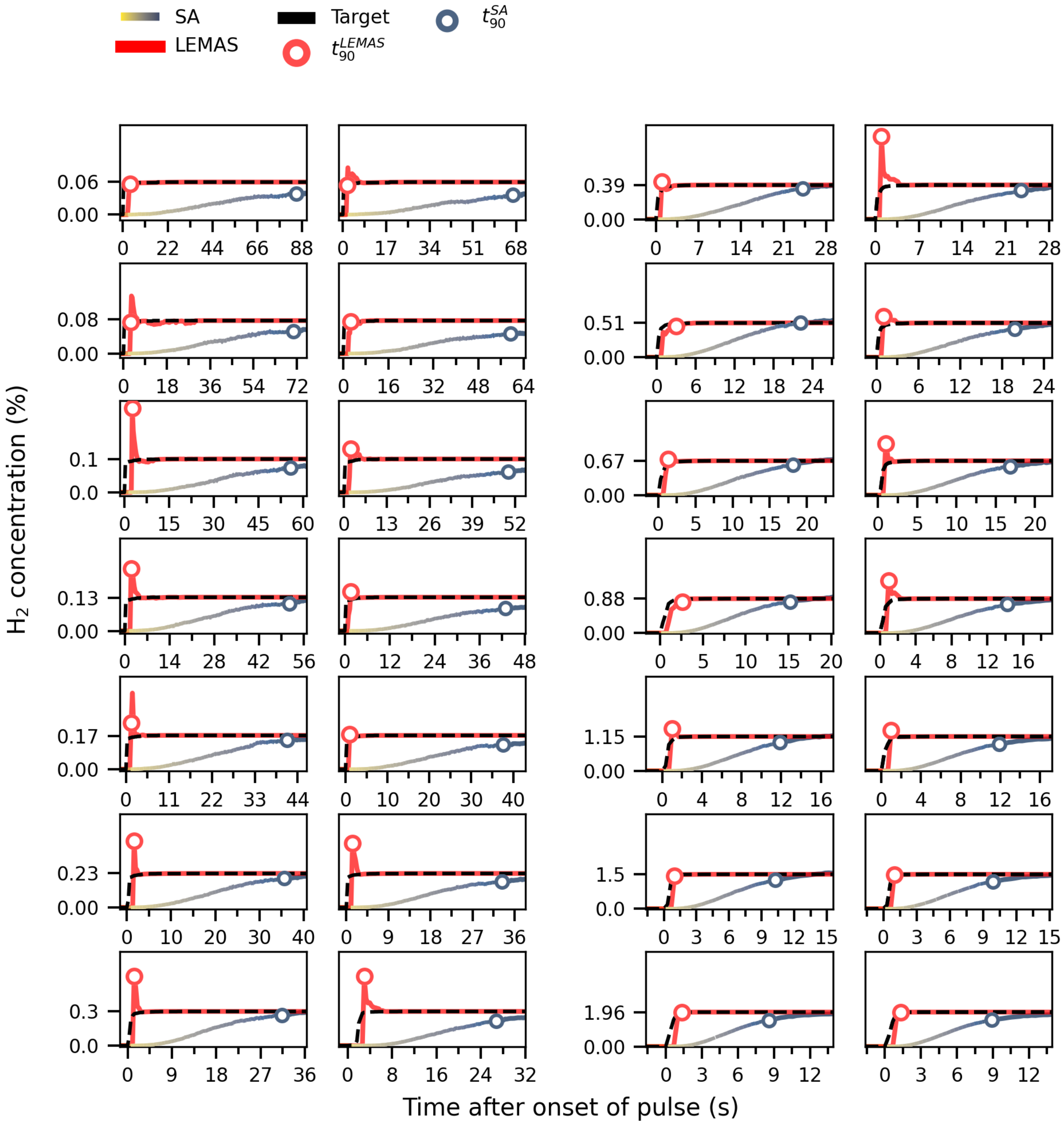

Figure S17: **Comparison of LEMAS and SA for step-wise increases in test data.**

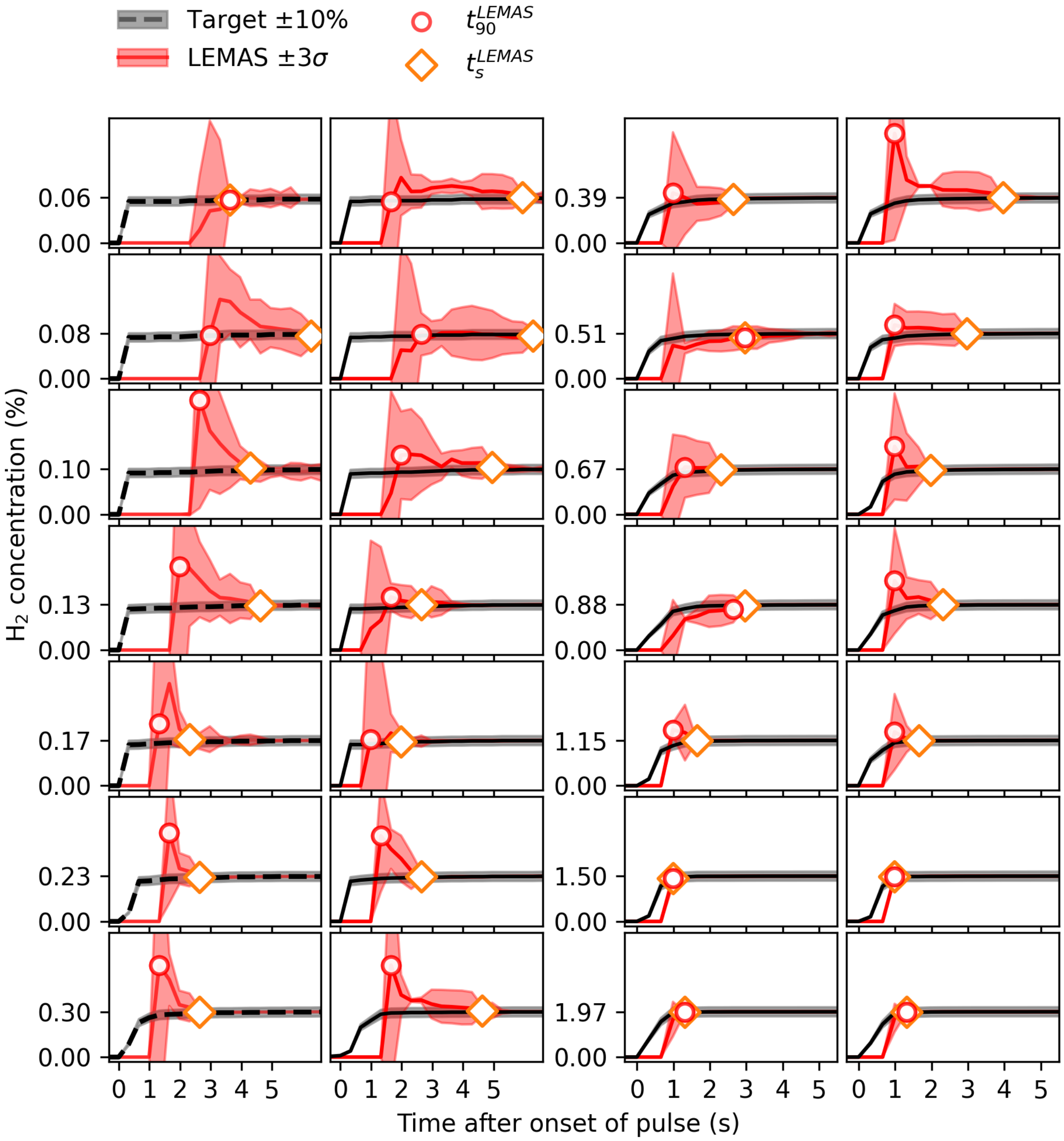

Figure S18: **Initial prediction of mean and standard deviation by LEMAS for step-wise increases in test data.**

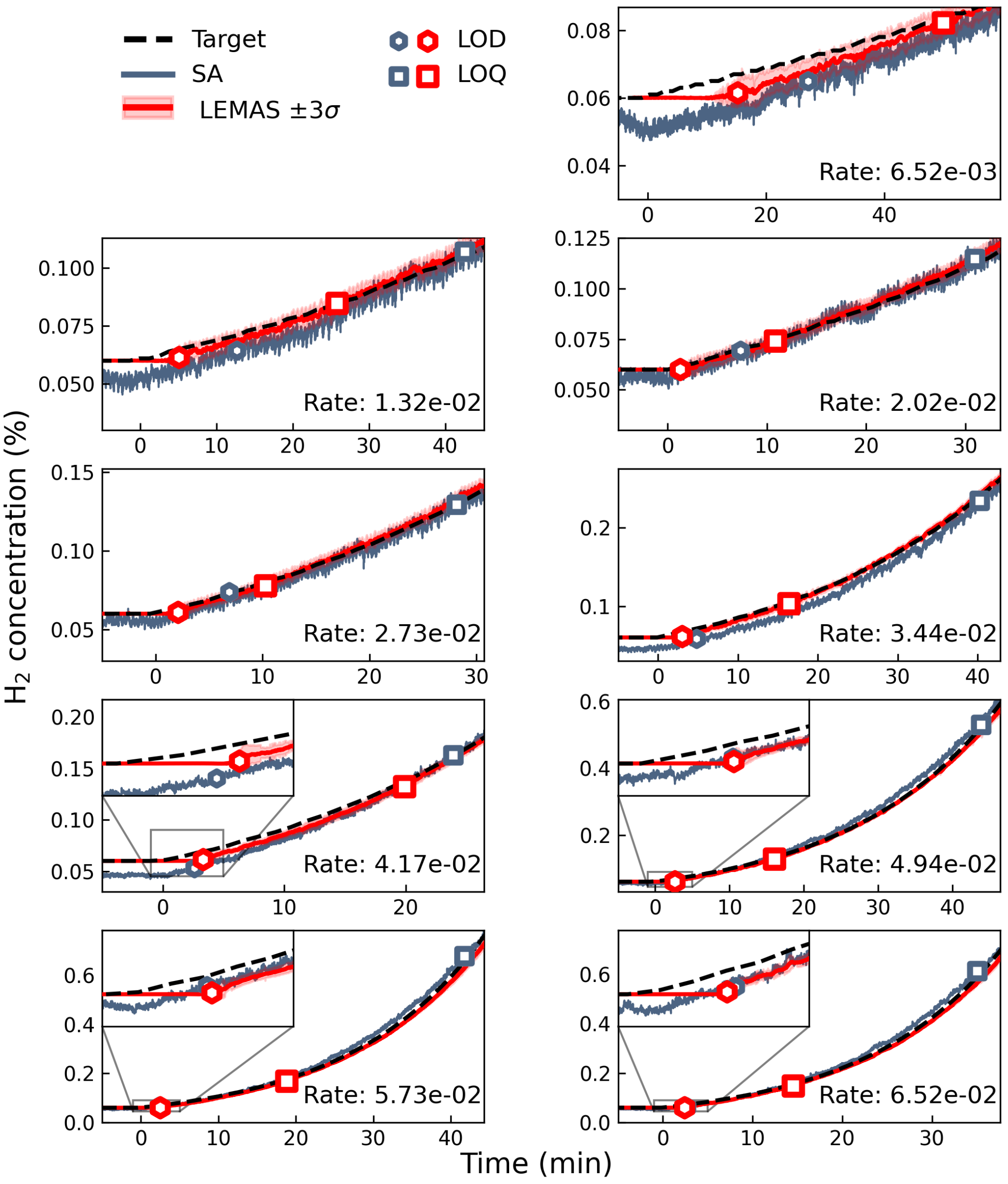

Figure S19: **Comparison of LEMAS and SA for exponential leak rates in test data.**

Supplementary Tables

Table S1: **Hyperparameters used for all models.**

Parameter	Value
n_{enc}	4
n_{dec}	8
n_0	8
n_1	4
d_{model}	256
d_{ff}	512
num heads	8
head size	32
Temporal stride on long term memory	4
n_{mlp}	8
d_{mlp}	512
Dropout rate	0.1

Supplementary References

[1] Iwan Darmadi. *Polymer-Nanoparticle Hybrid Materials for Plasmonic Hydrogen Detection.* PhD thesis, Chalmers University of Technology, Gothenburg, Sweden, January 2021. Available at `https://research.chalmers.se/en/publication/521833`.

[2] Mingze Xu, Yuanjun Xiong, Hao Chen, Xinyu Li, Wei Xia, Zhuowen Tu, and Stefano Soatto. Long short-term transformer for online action detection. In *NeurIPS 2021*, 2021. URL `https://www.amazon.science/publications/long-short-term-transformer-for-online-action-detection`.
